# Comparison of pulsed and continuous electromagnetic field generated by WPT system on human dermal and neural cells

**DOI:** 10.1038/s41598-024-56051-z

**Published:** 2024-03-06

**Authors:** Romana Zahumenska, Bibiana Badurova, Miroslav Pavelek, Peter Sojka, Tereza Pavlisova, Pavol Spanik, Monika Kmetova Sivonova, Slavomira Novakova, Jan Strnadel, Erika Halasova, Michal Frivaldsky, Henrieta Skovierova

**Affiliations:** 1 Jessenius Faculty of Medicine in Martin, Biomedical Centre Martin, Comenius University in Bratislava, Mala Hora 4C, 036 01 Martin, Slovakia; 2 Department of Medical Biochemistry, Jessenius Faculty of Medicine in Martin, Comenius University in Bratislava, Mala Hora 4D, 036 01 Martin, Slovakia; 3 Department of Mechatronics and Electronics, Faculty of Electrical Engineering and Information Technologies, University of Zilina, 010 26 Žilina, Slovakia

**Keywords:** Wireless charging, Wireless power transfer, Electromagnetic field, Biocompatibility, Neural cell lines, Cytotoxicity, Oxidative stress, Cell morphology, Cell biology, Engineering

## Abstract

In recent decades, we have seen significant technical progress in the modern world, leading to the widespread use of telecommunications systems, electrical appliances, and wireless technologies. These devices generate electromagnetic radiation (EMR) and electromagnetic fields (EMF) most often in the extremely low frequency or radio-frequency range. Therefore, they were included in the group of environmental risk factors that affect the human body and health on a daily basis. In this study, we tested the effect of exposure EMF generated by a new prototype wireless charging system on four human cell lines (normal cell lines—HDFa, NHA; tumor cell lines—SH-SY5Y, T98G). We tested different operating parameters of the wireless power transfer (WPT) device (87–207 kHz, 1.01–1.05 kW, 1.3–1.7 mT) at different exposure times (pulsed 6 × 10 min; continuous 1 × 60 min). We observed the effect of EMF on cell morphology and cytoskeletal changes, cell viability and mitotic activity, cytotoxicity, genotoxicity, and oxidative stress. The results of our study did not show any negative effect of the generated EMF on either normal cells or tumor cell lines. However, in order to be able to estimate the risk, further population and epidemiological studies are needed, which would reveal the clinical consequences of EMF impact.

## Introduction

Innovation, productivity and technological progress in the last century affect the functioning of the economy and, to a large extent, society as a whole. The external factors of the environment, that surrounds us, participate in pathological biological processes, thereby affecting the homeostasis of cells and maintaining the stability of the intracellular environment. Biophysical stimuli further affect cell signaling pathways, mitotic activity, DNA synthesis, embryogenesis, regeneration, gene expression, cell migration, and others^1–4^.

One of these external physical stimuli affecting organisms is the ubiquitous electromagnetic field (EMF). Data regarding the effects of EMF on cell biology are mixed and varied^5^. EMF has found use as a therapeutic tool in the treatment of bone defects, similar to wound healing^6–8^, on the other hand, it has also been associated with cases of cell death and apoptosis^9^.

One of the most used types of EMF is radio-frequency EMF (RF-EMF), which is classified at a frequency of 100 kHz–300 GHz. Devices emitting RF-EMF are used on a daily basis in almost all areas of ordinary and working life—welding machines, induction heaters, television and radio stations, microwave ovens, mobile phones, 5G telecommunications networks and others^10^. This technological development has brought with it many unanswered questions. Even if RF-EMF generating devices are classified as safe and satisfy current safety standards, what is their long-term impact? Is the daily use of these devices over the course of several decades indeed harmless? In 2011, the International Agency for Research on Cancer (IARC) classified RF-EMF as “potentially” carcinogenic to humans [Group 2B]^11^. However, in 2015, the Scientific Committee for Emerging and Newly Identified Health Risks (SCENIHR) adopted the opinion that the use of mobile phones with a range of RF-EMF exposure did not demonstrate an increased risk of developing oncological diseases in the head and neck area (and thus also brain tumors)^12^.

An extensive study of the effect of RF-EMF in animal models (more than 10,000 rats at a cost exceeding 5 million euros) was carried out at the Ramazzini Institute by the group of Soffritti et al. However, they were of such concern that the advisory committee recommended that IRAC reconsider the RF-EMF classification^13,14^. The committee suggested raising the risk to a “probable” carcinogen [Group 2A] and even “carcinogenic to humans” [Group 1]^15^.

Wireless devices used every day are constantly being innovated. New device models respond to new technological protocols and change their exposure scenarios therefore it is necessary to react. It is well known that external EMF generates electromagnetic waves that are absorbed by the living system, so possible health risks, effects or safety must be constantly identified. An attractive test model is represented by in vitro cell cultures, which are relatively cheap, easily available, sufficiently biologically characterized, and established.

The group of Gorski et al. observed morphological and cytophysiological changes in selected lines of normal and cancer human cells under the influence of RF-EMF. The results of the study indicate that EMF exposure caused a significant decrease in fibroblast viability and a significant increase in (prostate) cancer cells. However, cell lines were exposed to RF-EMF at a frequency of 2.5 GHz^16^. Several other studies focused on the development of oncological diseases of the central nervous system. The reason is the causal link between RF-EMF exposure and the occurrence of brain neoplasms. These have been recorded by several epidemiological studies because it is a specific organ, formed by highly sensitive nerve cells, with the highest specific absorption rate (SAR) of electromagnetic waves^17^. The group of Zielinski et al., observed the effects of exposure to pulse-modulated RF-EMF on apoptosis, autophagy, and oxidative stress in human neuroblastoma and mouse microglial cells. The results of the study demonstrated that exposure to RF-EMF (935 MHz, 4 W/kg) did not induce apoptosis in SH-SY5Y and microglial cells. It also suggests that short-term RF-EMF at SAR levels accepted by today's safety limits can cause autophagy and oxidative stress, with the effect depending on the cell type and duration of exposure^18^. Saliev et al.^19^ appropriately point out that although the biological effects of non-ionizing electromagnetic fields induce several pathophysiological mechanisms in cells (e.g. apoptosis, breaking points in DNA, etc.), these effects could be effectively used in therapy and treatment if they are correctly understood and interpreted.

Currently, there is a lack of sufficient consistent knowledge about possible risks and connections. The aim of this article is to study the biocompatibility of EMF and generated electromagnetic waves on human cell lines. The first cell line is human dermal fibroblasts (HDFa), which represent healthy cells. Dermal fibroblasts are isolated from the patient's skin, which serve as a durable, protective barrier, and are the first to be exposed to EMF. Other cell types tested are nerve cells, which are quite sensitive. Normal human astrocytes (NHA) represent a group of healthy cells. The other two types are neuroblastoma (SH-SY5Y) and glioblastoma (T98G) tumor cell lines. We tested two different EMF frequencies 87 kHz, resp. 207 kHz, with an operating intensity of 1.4–1.7 mT, resp. 1.3–1.5 mT and a transmitting power of 1.05 kW, resp. 1.01 kW. We observed the effect of EMF on cellular compactness at several biological and molecular levels. We compared control groups with groups exposed to EMF (pulsed and continuous exposure) at (a) morphological level (morphological changes, adherence ability) by bright field microscopy, (b) monitoring cytoskeleton remodeling by fluorescence microscopy, (c) cell viability by spectroscopy, (d) cytotoxic effect determined by flow cytometry, (e) genotoxic effect and DNA damage using fluorescence microscopy, (f) cellular oxidative stress using luminescence measurement. These analyzes could offer consistent findings and offer further insight into the safe use of newly developed prototype wireless charging systems.

## Material and methods

### Controlled environment incubator

**Figure 1:**
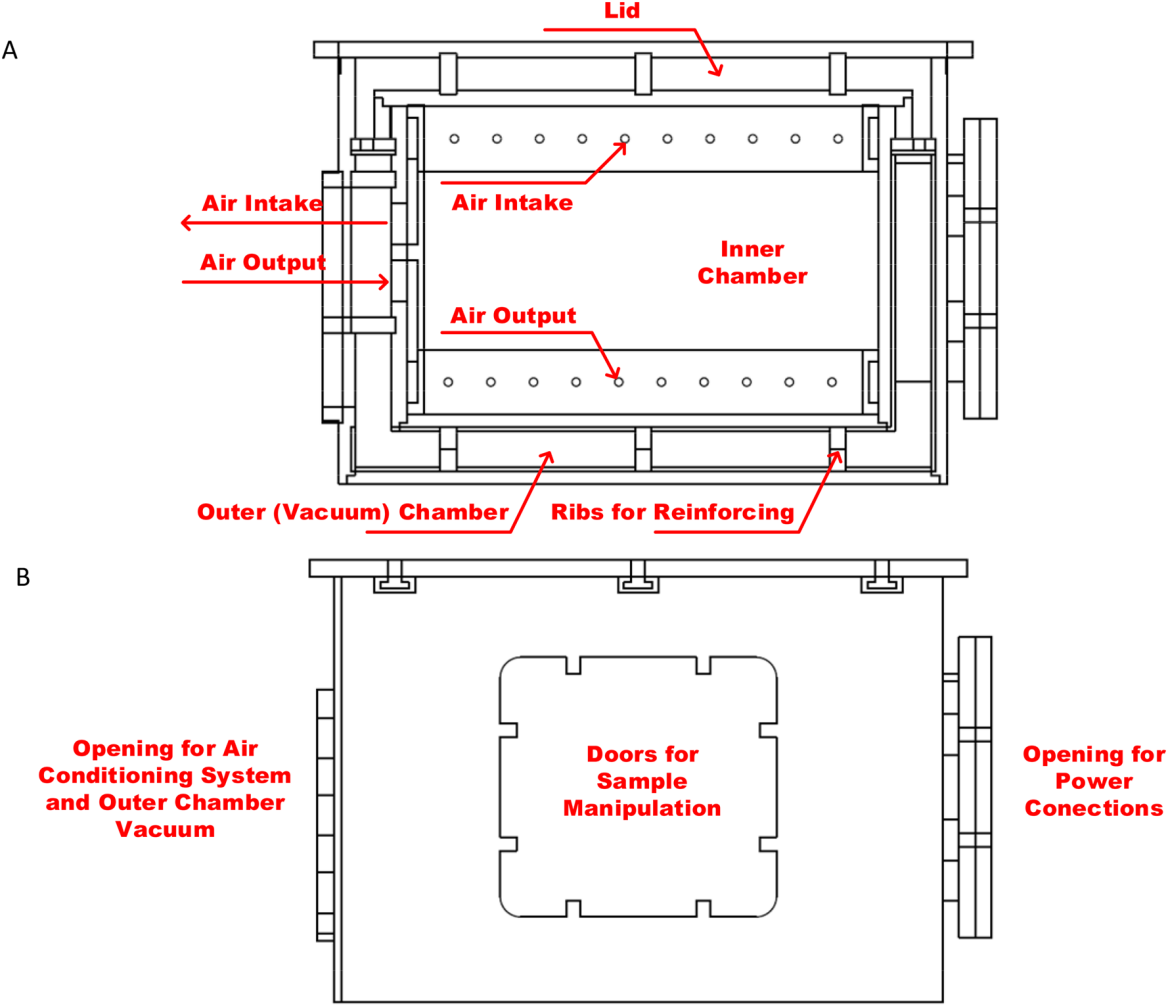
The scheme of thermo incubator (**A**)—sectional side view, (**B**)—side view.

Because in vitro cultivated human neural cells need to maintain a steady temperature within the EMF exposure, a particular experimental incubator is needed. Figure 1 depicts the geometrical layout of the suggested incubator, which considers the designs of two chambers: an interior temperature-controlled chamber and an exterior vacuum chamber.

It is necessary for the test temperature to be relatively near to the body temperature of a human. As a result, the incubator is using a heating system, and the temperature is controlled to stay between 35.5 and 37 °C. The system's material is 15 mm acrylic glass, and to withstand vacuum, the outer chamber is strengthened by ribs that have holes in them. The three air-sealed openings on the incubator are for the air inlet/outlet and vacuum for the outer chamber, the power connections for the WPT system, and the opening for handling biological samples. Since each of the three apertures is a separate component of the incubator system, they can all be fully configured. There are input air apertures around the lower section of the incubator’s inner chamber and output air ports around its upper part. To enable the WPT system to be inserted, the incubator’s upper section, known as the “Lid,” can be opened (this part is also air sealed). The 3D printed screws are used to attach all of the air-sealed components to the incubator. It is anticipated that in the future, the incubator environment will also include CO_2_ level control.

**Figure 2:**
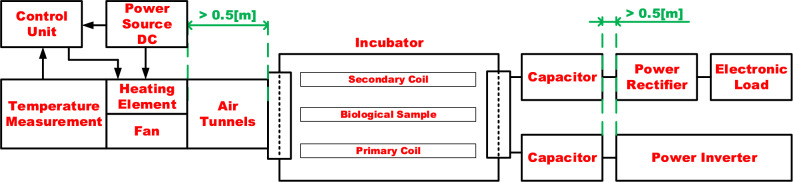
Block diagram of WPT measurement within the incubator with air-conditioning system.

In order to ensure that neither the air conditioning system nor the assessment of the WPT influence on the samples are impacted, Fig. 2 illustrates that the air heating is carried out in an exterior chamber for air conditioning, which is located 50 cm from the incubator’s inner chamber. A 40 mm server located on the system's inlet side creates the airflow inside the air conditioning system. The air conditioning system's outlet switches high current through power resistors to adjust the air temperature, which is then measured in a small compartment at the end of the system. The temperature regulation algorithm uses an Arduino Nano µC as the primary temperature sensor and operates in hysteretic mode to maintain the temperature within a predetermined range.

### Wireless power transfer system

**Figure 3:**
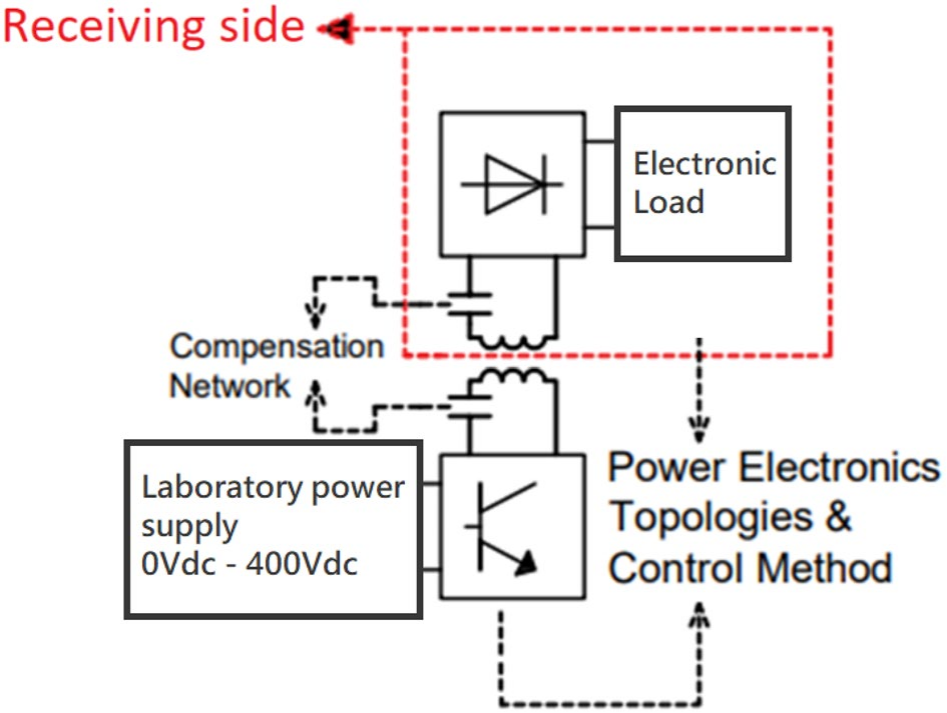
Block diagram of the configuration of WPT system.

Figure 3 illustrates the WPT system configuration for the EMF exposure experiment. It is made up of a primary side high-frequency inverter that feeds a primary side transmitting coil with a compensating capacitor, as well as a laboratory power supply unit. The receiver, compensating capacitor, high-frequency rectifier, and programmable electronic load make up the receiving side, or secondary side. The finite element method (FEM) model is used to identify the EMF intensities, whilst the PSU (Power Supply Unit) Input voltage of the WPT system modifies the EMF's intensity^20^.

**Figure 4:**
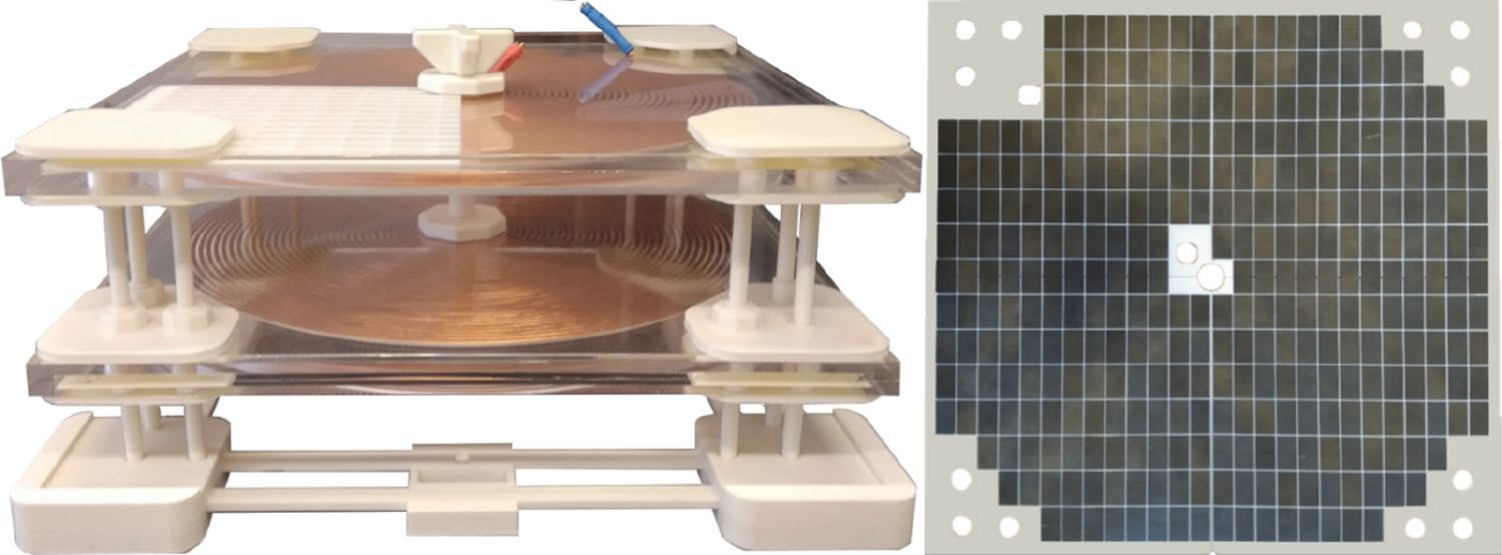
System of coupling coils with adjustable transfer distance (left) and reconfigurable magnetic shielding (right).

The suggested WPT system’s operating parameters are in line with the application of neural human cells^21^. It means, that transmitted power level relevant electromagnetic field quantities are set, so that their values refers to the application with the neural cells. If, for example, the human body was considered as a whole, then the EM field would be set to significantly higher levels. In other words, the system can be set in such a way that it corresponds to the investigation of the influence of exposure at different scales corresponding to the structures of the human body (neural cells, skin cells etc.). Reconfigurable in nature, the coupling coil system was created with the possibility of the adjustable power transmission distance and electromagnetic shielding in mind. The coil matrices are positioned on the construction so that the mutual distance of the coils can be readily changed between 0.05 and 0.25 m (Fig. 4). As was described earlier, the magnetic intensity for the selected exposing component should be at the maximum level of 1.2 mT at the frequency of EMF 87 kHz. To identify the distribution of the EMF around the designed coupling coil system, FEM analysis was provided to identify Input/Output parameters of WPT system. The analysis is reconfigurable according to the use of shielded, or non-shielded coupling coil system. The use of the shielding is important to reduce negative impacts of radiated EMF above the system of the coils. Proposed shielding system also enables investigation of the various geometrical arrangement on the EMF distribution^20–22^.

### Simulation analysis of electromagnetic field

Before doing studies involving electromagnetic field exposure, the magnitudes of the magnetic field must be determined. There are a few methods for determining EMF intensities; the first is experimental, and the second involves using a validated simulation model. We have used the second option, which is the extremely accurate simulation model of the suggested WPT system^20^, since the laboratory equipment for experimental measurement of EMF radiation may be complicated and costly.

**Figure 5:**
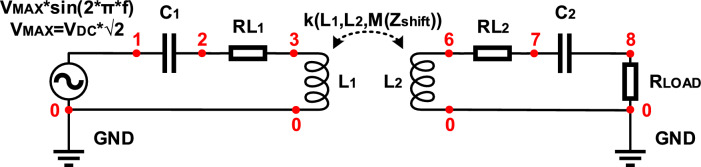
Electrical circuit definition within electrical domain of the FEM simulation model.

**Figure 6:**
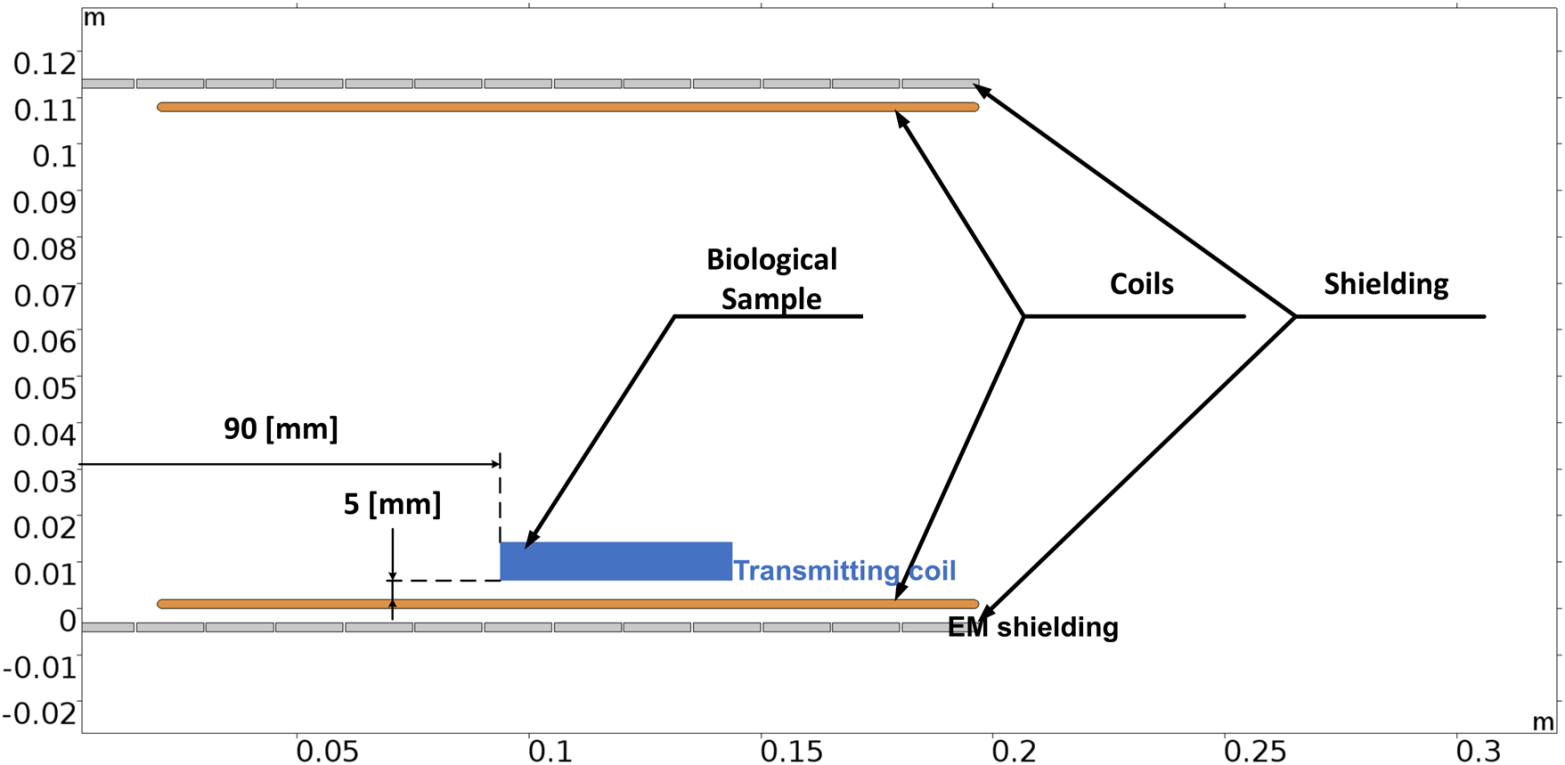
The placement of the biological samples in the WPT system during exposure (displayed in vertical symmetry).

Figure 5 depicts the equivalent circuit that was utilized to determine the EMF intensities. The electrical and magnetic domains are taken into account by the model, and Table 1 provides electrical circuit settings. The positioning and arrangement of the measuring sites inside the setup are shown in Fig. 6.

**Table 1:** Settings of the electronic circuit simulation model.

Circuit element	Value	Point (+)	Point (−)
Ground	GND	0	0
Voltage source	VMAX*sin(ωt)	0	1
Capacitor 1	1/(ω^2*comp1.mf.LCoil_1) [F]	1	2
Resistor 1	RCoil1 [Ω]	2	3
External I vs. V1	Coil voltage (mf3/coil1)	3	0
External I vs. V2	Coil voltage (mf3/coil2)	0	6
Resistor 2	RCoil2 [Ω]	6	7
Capacitor 2	1/(ω^2*comp2.mf2.LCoil_1) [F]	7	8
Load	RLOAD [Ω]	8	0

With the simulation analysis, the Magnetic flux was identified for the area, where the biological sample will be located. The biological samples will be located within the area between transmitter and receiver approximately 5 mm above transmitting coil and 90 mm from the coil axis. In this specific point is also defined maximal value of magnetic flux density, which will be applied to the sample.

**Figure 7:**
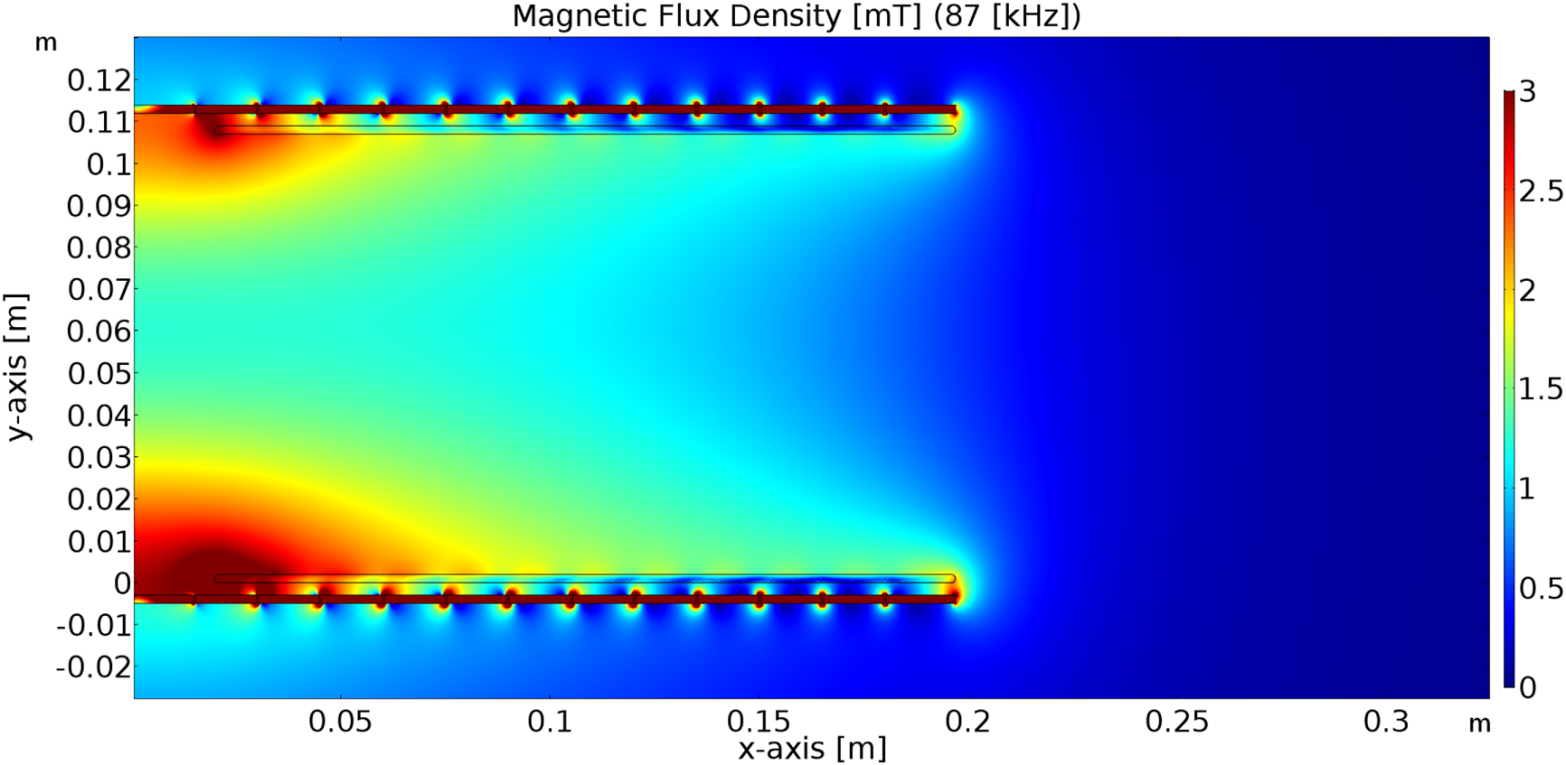
Simulation results of the distribution of magnetic induction within system of coils (operating frequency, 87 kHz; transmitting power, 1050 W).

**Figure 8:**
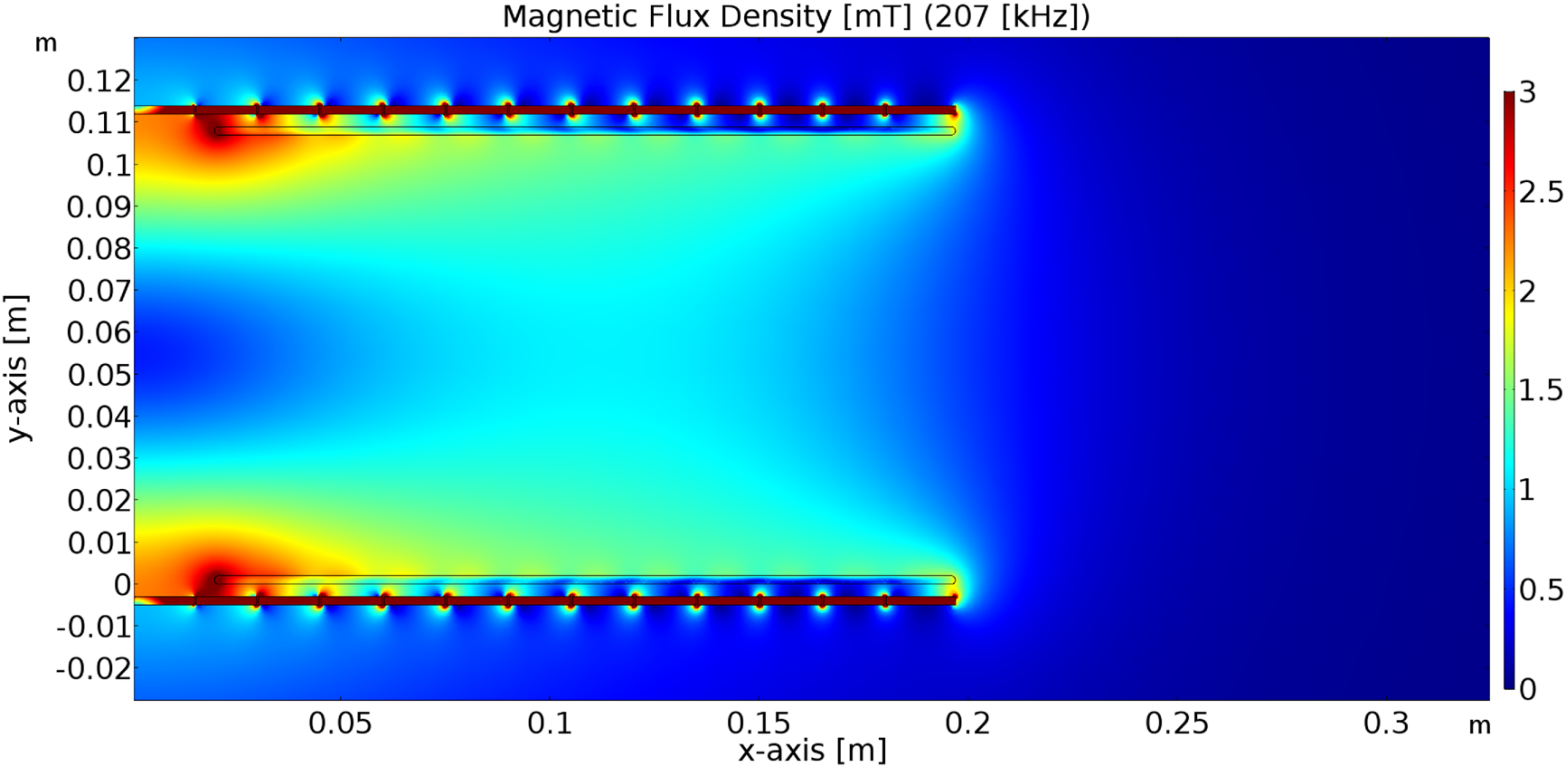
Simulation results of the distribution of magnetic induction within system of coils (operating frequency, 207 kHz; transmitting power, 1010 W).

To identify the range of magnetic flux density, which are required for the exposure of the biological samples, the parametric simulation analyses were performed identified target value of magnetic induction. The example of the simulation result is shown on Figs. 7 and 8. As can be seen in Fig. 7, the range of the magnetic flux density applied to the sample will be approximately 1.4–1.7 mT, while Input/Output parameters of WPT system for experiments are identified as follows, Input DC voltage 213 V; Resistive load 48 Ω; Main Resonant Frequency 85 kHz—switching frequency of high-frequency inverter; and Higher Resonant Frequency 87 kHz.

For Input/Output parameters of WPT system defined as follows, Input DC voltage 213 V; Resistive load 38 Ω; Main Resonant Frequency 177 kHz—switching frequency of high-frequency inverter; and Higher Resonant Frequency 207 kHz, the range of the magnetic flux density applied to the sample will be approximately 1.3–1.5 mT, this can be seen in Fig. 8.

**Figure 9:**
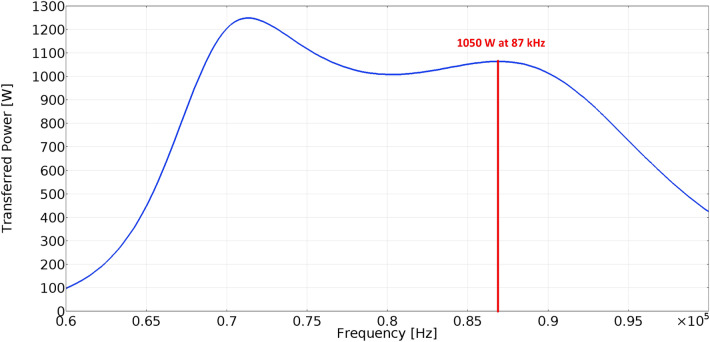
Frequency characteristic of the WPT system for the EMF distribution from Fig. 7.

**Figure 10:**
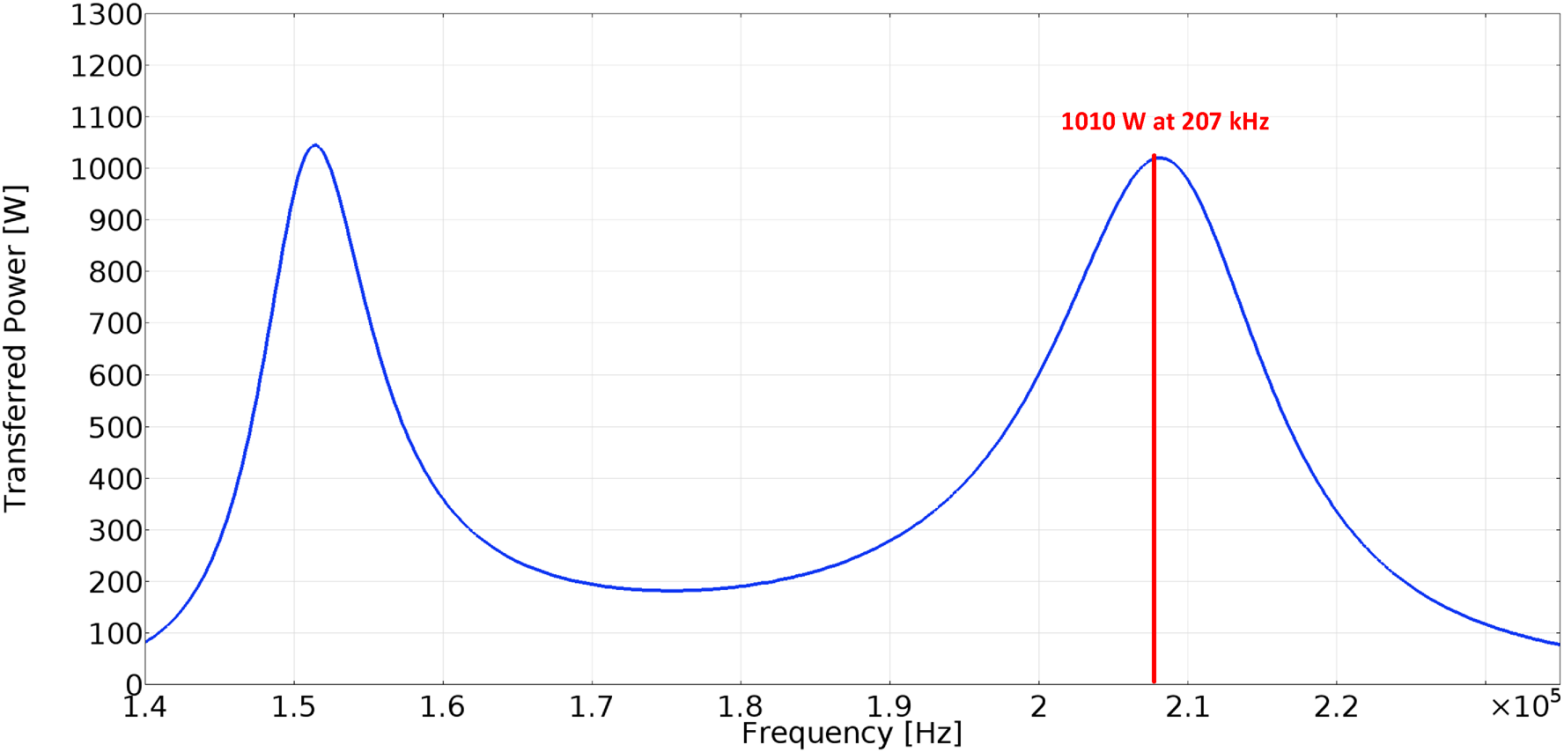
Frequency characteristic of the WPT system for the EMF distribution from Fig. 8.

Investigating the frequency characteristic for above parameters Figs. 9 and 10 represents dependencies of the transmitted power on the switching frequency of the inverter. The value of power transfer for investigated EMF exposure is approximately 1.05 kW and 1.01 kW respectively.

Due to the usage of neural human cells, it must be highlighted that the WPT system's aforementioned parameters indicate scaled exposure. When examining diverse exposure samples, such as human tissues or larger biological structures, it is necessary to evaluate higher EMF intensities. This can be achieved by varying the WPT system’s Input/ Output settings.

### Cell cultures and culture conditions

Four different commercially available human cell lines were used in this study. Two lines represented healthy cell types. Human dermal fibroblasts (HDFa) (Gibco, USA), were grown in DMEM:F12 (1:1) + Glutamax (Gibco, USA) culture medium, supplemented with 10% FBS (Biosera, GB) and 100 I.U./mL of penicillin and 0.1 mg/mL of streptomycin (Biosera, GB). Normal human astrocytes (NHA) (ScienCell, Carlsbad, CA, USA), were cultivated in Astrocyte Media (ScienCell, USA) supplemented with 2% FBS (ScienCell, USA), 1% AGS (100×; ScienCell, USA) and 100 I.U./mL of penicillin and 0.1 mg/mL of streptomycin (Biosera, GB).

The other two cell lines represented tumor cell types. Human neuroblastoma cell line (SH-SY5Y) (ECACC, UK), was cultivated in DMEM:F12 (1:1) + Glutamax (Gibco, USA), supplemented with 10% FBS (Biosera, GB) and 100 I.U./mL of penicillin and 0.1 mg/mL of streptomycin (Biosera, GB). Human glioblastoma cell line (T98G) (ECACC, UK), was cultivated in DMEM high glucose medium (Sigma-Aldrich, USA), supplemented with 10% FBS (Biosera, GB) and 100 I.U./mL of penicillin and 0.1 mg/mL of streptomycin (Biosera, GB).

Cells were cultivated at standard conditions (5% CO_2_, 37 °C, humidified atmosphere). HDFa and T98G were plated at the density of 6.3 × 10^3^ cells/cm^2^, NHA and SH-SY5Y were plated at the density of 9.4 × 10^3^ cells/cm^2^.

### Exposure of cell cultures

Before exposure to EMF, cells were cultivated under standard culture conditions to an initial confluence of 35–45% in flask or microtiter plates. For details, see Skovierova et al.^23^. Subsequently, the cells were divided into three groups: (1) control group (non-exposed); (2) group exposed to pulsed EMF (6 × 10 min); and (3) group exposed to continuous EMF (1 × 60 min). Both exposed groups were placed on the coil. Cells affected by pulsed EMF have 10 min pause between pulses. These parameters were set based on the scale limits of the prototype.

The control group of each cell line, unexposed cells, were cultivated out of reach of any artificial EMF in at 37 °C. After the exposure period, cells were cultivated for an additional 30 h under standard conditions before further analyses. During this period, at least one new generation of cells was obtained. Therefore, a potentially harmful effect of EMF on the samples or late effects of EMF exposure on cells can be considered.

### Cell morphology

Cell morphology was monitored at each cell line before exposure; immediately after exposure; and 30 h after regeneration under standard cultivation conditions by light microscope under inverted phase contrast (Optika XDS-2, Italy), magnification 100×.

### Immunocytochemistry

Immunocytochemical staining was done similarly as Skovierova et al.^20^. Briefly, samples after EMF exposure and 30 h regeneration washed with DPBS followed by 4% paraformaldehyde (Cell Signaling Technologies, USA) fixation for 30 min at room temperature. After washing, cells were incubated with Alexa Fluor® 488 Phalloidin (Cell Signaling Technologies; 1:1000 in a solution of 1% BSA in DPBS) for 80 min. Consequently, the samples were labeled with DAPI dye (Sigma-Aldrich, USA) for 10 min at room temperature in the dark. Cells were washed three times with DPBS and analyzed with a fluorescence microscope (WiScan®, Hermes IDEA Bio-Medical, Israel), magnification 100×.

### Cell viability assay

An assay (also known as MTT assay) was performed to analyze the possible cytotoxic effect of EMF on cell viability. MTT is a biochemical screening test that is based on spectrophotometric measurement of the amount of the product of the succinate dehydrogenase enzymatic reaction, the enzyme of the basic energy metabolism of the cell, the Krebs cycle. The measured absorbance intensity is directly proportional to the cell viability. Thus, as the absorbance value increases, the amount of metabolized MTT also increases, which is a direct indicator of cell viability.

Cells of each cell line were seeded into 96-well microtiter plates in hexaplets. After exposure, the cells were divided into two groups: (a) those were analyzed immediately, without the possibility of regeneration, to note the potential direct negative effect of EMF on the cells, and (b) those regenerated for 30 h under standard culture conditions, during which there should have been cell doubling and the production of a new daughter generation. It is important for a better comparison of the cumulative effect of EMF between the exposed group compared to the control, non-irradiated group.

We tested two different frequency settings of 87 kHz, resp. 207 kHz, while maintaining the same intensity and power of the device 1.05 kW, resp. 1.01 kW and 1.4–1.7 mT, resp. 1.3–1.5 mT. The tested group of cells immediately after irradiation as well as the group of cells after 30 h of regeneration were washed with DPBS (Biosera, GB) at the beginning of the viability test. Further were cells incubated in a fresh culture medium containing the addition of MTT (Sigma-Aldrich, USA) for another 5 h at 37 °C in a humidified atmosphere. Then, 5% SDS (w/v; Sigma-Aldrich, USA) was added to dissolve the formazan salt. After 18 h of incubation, colorful changes were measured spectrophotometrically at 540 nm using an Epoch Microplate Reader (BioTek, Germany). Relative cell viability was determined as the ratio of the O.D. (Optical Density) value of formazan produced by irradiated cells to the O.D. value of formazan produced in the control group and expressed as a percentage of control. O.D. of control cells was taken as 100%. All experiments were performed at least three times. MTT assay results were expressed as mean ± standard deviation (SD) of 6 replicates as indicated. Statistical analysis was performed using one-way analysis of variance (ANOVA) and the significance of the difference between means with p < 0.05 was considered statistically significant.

### Annexin assay

The Annexin assay is a fluorescent analysis of basic cellular processes, categorizing cells into four groups (live cells, cells in the early phase of apoptosis, cells in the late phase of apoptosis and dead cells). Through flow cytometry and the use of fluorescently labeled markers, Annexin V with a fluorescently conjugated APC label, which specifically binds to phosphatidylserine (a phospholipid typical of the inner side of the plasma membrane) and 7-AAD (a fluorophore penetrating the nucleus of dying cells, binding to DNA) to identify apoptotic and necrotic cells. Dying cells have an increased distribution of phosphatidylserine on the outside of the plasma membrane, which enables the binding of the fluorophore to the cells and subsequently its positivity detected by flow cytometry. Briefly, after 30 h of regeneration, a single cell suspension was prepared and resuspended in Annexin V binding buffer (BioLegend®, USA) and incubated with Annexin V dye. Subsequently, the staining solution was added to the samples of 7-AAD viability, samples were incubated for 15 min in the dark at room temperature. After the addition of Annexin V binding buffer, fluorescently labeled cells were analyzed by flow cytometry (FACS Aria™ Cell Sorter, BD Bioscience, USA).

### Comet assay

The method is a combination of DNA electrophoresis and fluorescence microscopy which is used for the quantification and analysis of DNA damage of individual cells. It got its name from the comets formed during electrophoresis. The head of the comet contains intact DNA and the tail of the comet indicates damaged DNA and its fragments.

After irradiation, cells were allowed to regenerate for 30 h. Then, cells were trypsinized by TrypLE Express (Thermo Fisher Scientific, USA), centrifuged (450 × g, 3.5 min), and subsequently resuspended in DPBS. The number of cells in suspension was counted on a Cell Counter (BioRad, USA) and a precisely determined number of cells was used for Comet assay analysis. Before analysis, the individual tested groups were fixed in a volume of 250 µL in 0.7% LMA (37 °C) and applied to a microscope slide with a rough surface (1% NMP agarose) in the number of approximately 100,000 cells. The samples were covered with a coverslip those were removed after agarose solidification. Slides with fixed cells were immersed in a cuvette with a lytic solution (10 mmol/L Tris–Cl; 100 mmol/L Na_2_EDTA; 2.5 mol/L NaCl; pH 10; 1% Triton-X100) and cell lysis proceeded for 1 h at 4 °C. The slides were alkaline treated in electrophoretic buffer (1 mmol/L Na_2_EDTA; 0.3 mol/L NaOH; pH 13) for 40 min, 4 °C. The electrophoretic conditions were 25 V, 300 mA, 30 min, and 4 °C. The samples were neutralized by buffer (0.4 mol/L Tris–Cl; pH 7.5) for 3 × 5 min, 4 °C. Samples were stained with DAPI (1 µg/mL) and analysis of DNA stability was performed by fluorescence microscope (Olympus IX73, Japan) magnification 100×.

### Oxidative stress analysis

The electromagnetic field generated by wireless energy transfer can affect the oxidative status of cells and negatively affects living organisms by increasing the production of free radicals (mainly oxygen radicals, ROS). The impact can occur at several levels, both subcellular and cellular. The GSH assay is a luminescence assay aimed at the detection and quantification of glutathione (GSH) in cells. ROS or drug interactions often cause a decrease in GSH levels, also through their reaction with the thiol group. Measuring the level of GSH is important for the assessment of toxicological reactions that can promote oxidative stress and lead to apoptosis and cell death. If the GSH level is reduced, the capacity of the cells for detoxification is also reduced.

In the first step, we measured cell viability using the CellTiter-Fluor™ Cell Viability Assay (Promega, Germany) followed the manufactured protocol. Briefly, CellTiter-Fluor® Reagent was added to analyzed cells in a 96-well plate, mixed, and incubated for 30 min at 37 °C. As background, the cultivation media with Reagent were used. The fluorescence signal was measured at λ 380–400 nm_Ex_/505 nm_Em_ using GloMax® Discover Multimode Detection System (Promega, Germany). Viability was measured in triplicates of each cell line and each analyzed group.

In the next step, the oxidation status was estimated by the GSH detection test (GSH-Glo™ Gluthation assay, Promega, Germany) which provides a simple and sensitive luminescent method. Briefly, after removing cultivation media, 100 µL of 1× GSH-Glo™ Reagent was added into each well in 96 well plate, mixed, and incubated for 30 min at room temperature. Then, 100 µL of Luciferin Detection Reagent into each well, mixed and incubated for 15 min at room temperature. Finally, the luminescence was measured using GloMax® Discover Multimode Detection System (Promega, Germany) to determine the total GSH level was calculated by extrapolation to the calibration curve of GSH. All experiments were performed three times. Results were expressed as mean ± standard deviation (SD) of triplicates. Statistical analysis was performed using one-way analysis of variance (ANOVA) and the significance of the difference between means with p < 0.05 was considered statistically significant.

## Results

**Figure 11:**
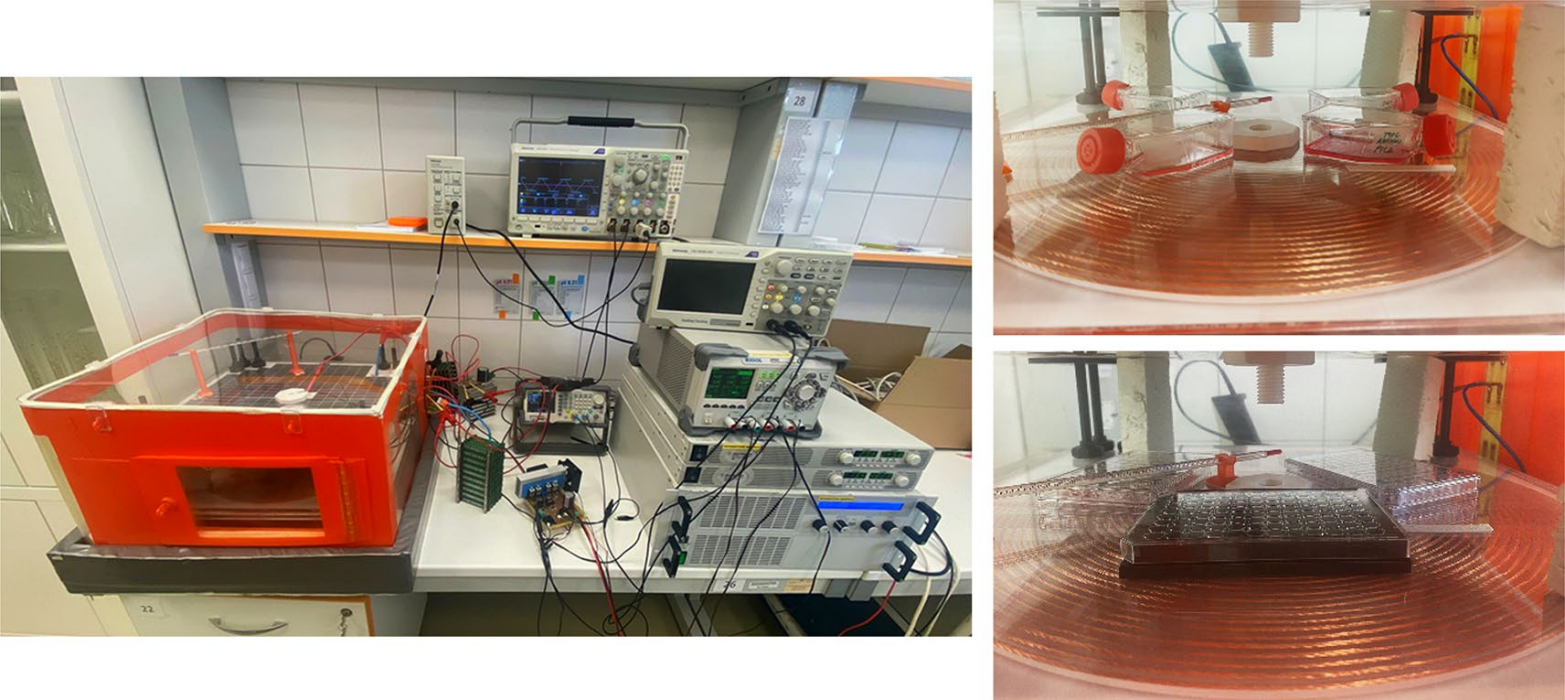
Experimental set-up of the wireless power transfer system (left) and detail of location of biological samples within coils coupling system (right).

Based on the simulation analyses, we have adjusted operational parameters (Input/ Output) of physical sample according to previously received results. The coupling coils system have been placed within thermo incubator and the biological samples (based on the Figs. 6, 7 and 8) have been placed between the coils (Fig. 11). The operational frequency of WPT system as well as the frequency of EMF was equal to 87 kHz (and 207 kHz respectively), transmitted power was 1.05 kW (and 1.01 kW respectively) and the value of magnetic induction within individual cells varied from 1.4 to 1.7 mT (and from 1.3 to 1.5 mT respectively) based on the location. Compared to the standards issued by ICNIRP 2010 (the limit is 27 µT), the experimental settings exceed the limit in range 48–63 times. These increased values of induction were set to test more extreme conditions, compared to the limit ones. We compared the effect of EMF exposure on various types of cells.

Comparing the effect of EMF on skin cells and neural cells is quite attractive. Skin fibroblasts, are quite resistant and are the first to be exposed, on the other hand, neural cells are very sensitive to any external stimuli and their regenerative capacity is very low. The coupling coil system was placed in a thermal incubator (maintained at a constant temperature of 37 °C) and the biological samples were placed on the coils, where the greatest EMF exposure was generated.

### EMF effect on cell morphology and cytoskeleton remodeling

**Figure 12:**
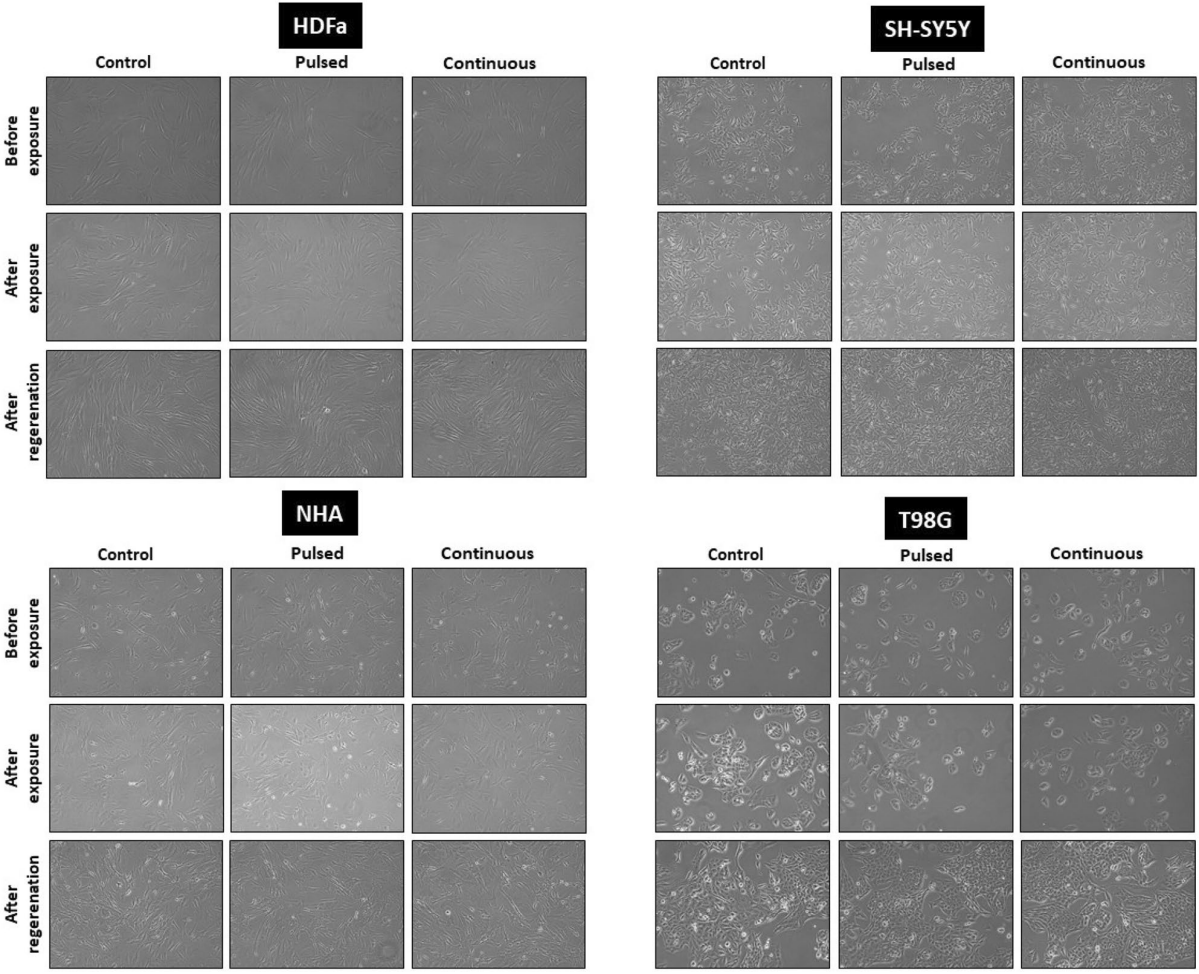
Cell morphology of human dermal fibroblasts (HDFa) and human neural cells (NHA, T98G, SH-SY5Y) analyzed by light microscopy. Cells before EMF exposure (1st line), cells directly after exposure (2nd line), and cells after 30 h of regeneration in standard culture conditions (3rd line). The control group for individual cell type (1st column), cells exposed to pulsed radiation (87 kHz, 6 × 10 min; 2nd column), and cells exposed to continuous radiation (87 kHz, 1 × 60 min; 3rd column). Representative images were taken by phase contrast at 100× magnification.

Under the set conditions, we did not observe changes in the morphology or in the ability of adherence in all four types of cells that were exposed to EMF (87 kHz, 1.4–1.7 mT, 1.05 kW) compared to the control groups (Fig. 12). The cells didn’t have inhibited mitotic activity. After regeneration for 30 h under standard culture conditions, we observed an increased confluence of cells from 30% (before irradiation) to 70–80% (after regeneration). Cell confluence as well as preserved morphology was comparable to the control group in all exposed cells. Cells cultivated in T25 flasks were processed for Comet and Annexin analysis after regeneration. Although the number of cells after passaging varied slightly between control and exposed samples. In general, the viability of the passaged cells was very high, 90–100% (HDFa 99–100%; NHA 95–98%; SH-SY5Y 90–94%; T98G 99–100%).

**Figure 13:**
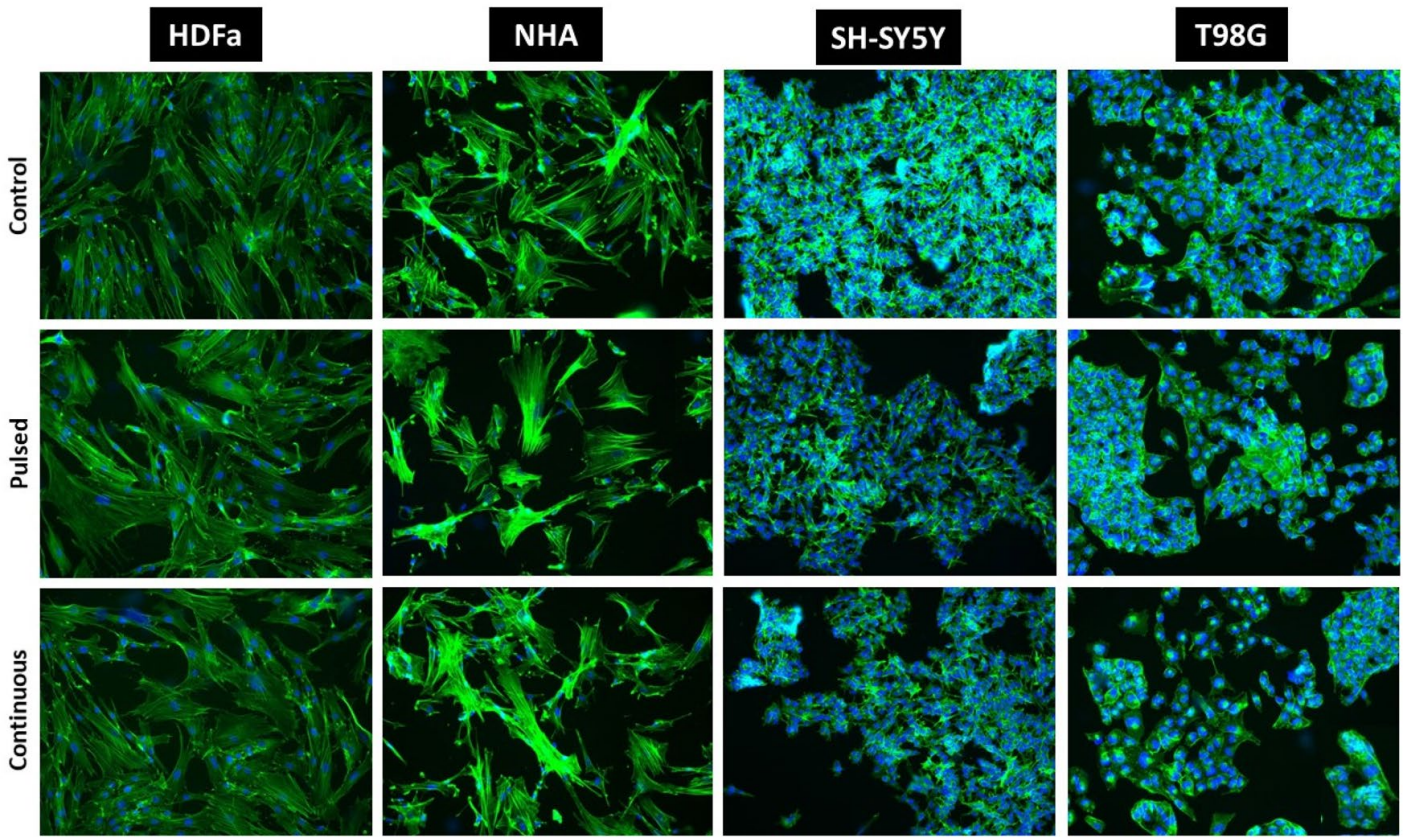
Immunocytochemical analysis recording cytoskeletal remodeling of human dermal fibroblasts (HDFa) and human neural cells (NHA, T98G, SH-SY5Y) analyzed by fluorescence microscopy. The control cells, not exposed to EMF (1st line), cells were exposed to pulsed radiation (87 kHz, 6 × 10 min, 2nd line), and cells exposed to continuous irradiation (87 kHz, 1 × 60 min, 3rd line). Representative images observing possible changes in the arrangement of actin filaments were taken after 30 h of regeneration under standard conditions at 100× magnification.

To analyze the intracellular space of the cell and its arrangement, the most appropriate imaging analysis is fluorescence microscopy. Immunocytochemical analysis (ICC) shows exactly what and how was reorganized inside the cell itself. With the help of a specifically labeled fluorescent antibody, Phalloidin, which selectively binds to the actin fibers of the cell cytoskeleton, it is possible to analyze cell shape, cytoskeleton remodeling, motility, division, and the movement of organelles. For better visualization, we use the blue DAPI dye, which intercalates into the DNA in the cell. Phalloidin, which we used in ICC analysis, will show the cell cytoskeleton in green (Fig. 13).

ICC showed no significant changes in cytoskeleton structure between exposed cells compared to control groups in normal cells (HDFa and NHA). HDFa retained their long, fibroid shape and fibrous structure, the cytoskeleton was dense and distinctly organized in parallel bundles. Similarly, NHA cells show a distinct fibrous cytoskeletal structure, organized in parallel bundles. The nuclei of HDFa and NHA cells are rounded with uncondensed chromatin. In both types of irradiated cells, there was no significant fragmentation of the nuclei into small apoptotic bodies, which could also be evidence of the negative effect of radiation on the cells. Likewise, the structure of the cytoskeleton is very compact and clearly defined in irradiated cells, no loose or significantly shortened fibers were observed. We did not observe differences in the groups of cells exposed to pulsed or continuous EMF radiation.

In the ICC analysis of tumor cells (SH-SY5Y, T98G), the amount of cytoskeleton is significantly lower compared to healthy cells of the control group. A large part of the cell is occupied by the nucleus itself (blue staining). The structure of the cytoskeleton is not strongly organized, as in healthy cells, and it is not clearly defined either. Tumor cells grow in groups, layered over each other. Nevertheless, the intensity, as well as the amount of green dye in the irradiated cells, is comparable to the control group. No apoptotic bodies or cells losing adherence are observed. The fact that the cells grow in clusters only mimics the natural properties that tumor cells exhibit.

### Effect of EMF on cell viability

**Figure 14:**
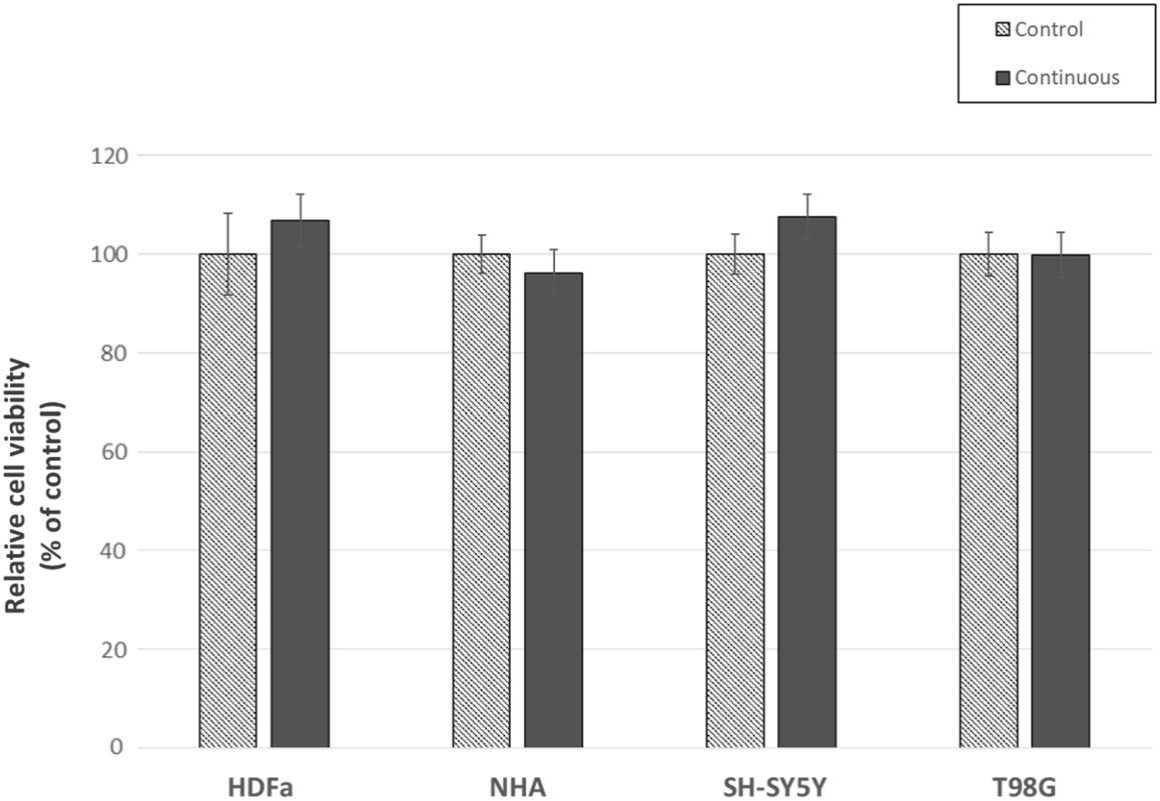
Effect of direct EMF exposure on cell viability of HDFa, NHA, SH-SY5Y, and T98G cells. Cell viability was analyzed by MTT assay. The cells were exposed to radiation with the following parameters: 87 kHz, 1.4–1.7 mT, and 1.05 kW for a continuous exposure time of 60 min. Analysis was performed immediately after EMF exposure, without a recovery period. The control group represents 100% viability. Data are mean ± SD. Data are representative of three independent experiments, each with 6 replicates.

**Figure 15:**
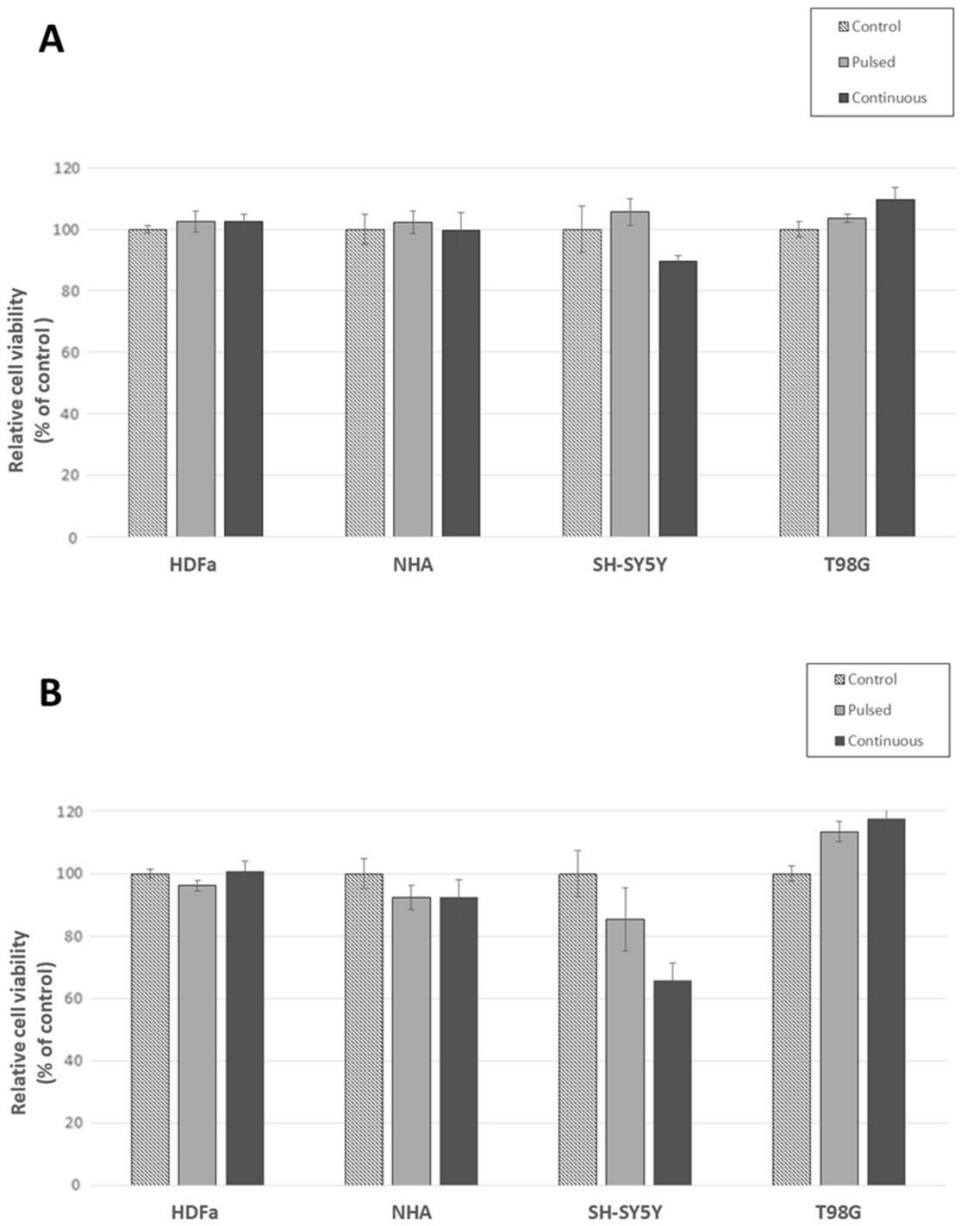
Effect of EMF exposure on cell viability of HDFa, NHA, SH-SY5Y, and T98G cells after 30 h regeneration. Cell viability was analyzed by MTT assay. (**A**) Cells were exposed to radiation of the following parameters (87 kHz, 1.4–1.7 mT, 1.05 kW) for pulsed EMF (6 × 10 min), and continuous exposure (1 × 60 min). The control group represents 100% viability. (**B**) The cells were exposed to radiation of the following parameters (207 kHz, 1.3–1.5 mT, 1.01 kW) for pulsed EMF exposure (6 × 10 min) and continuous exposure (1 × 60 min). The control group represents 100% viability. Data are mean ± SD. Data are representative of three independent experiments, each with 6 replicates.

We also analyzed the effect of EMF exposure on cell viability and basal energy metabolism using the MTT assay. After exposure, we divided all cell types into two groups: (a) samples analyzed immediately without the possibility of regeneration to record the potential negative effect of EMF (Fig. 14) and (b) samples allowed to regenerate for another 30 h under standard culture conditions (Fig. 15).

During regeneration, the creation of a new generation of cells should occur, and thus cell duplication. In this way, the cumulative effect of EMF radiation on the exposed group of cells could be better observed. At the same time, we were able to evaluate the results obtained from the exposed groups and compare them with the control, non-irradiated group. We also tested two different frequency settings (87 kHz and 207 kHz), while maintaining the same intensity and power of the device, and their effect on cell viability 30 h after regeneration.

Based on light microscopy observations, all four cell types maintained their morphological shape, while increasing confluence was observed in each type. By spectrophotometric analysis of the measurement of the color change of the solution, we converted the absorbance data to a value of 100%, which was represented by the control group of the given cell type.

When evaluating the MTT assay in a group of cells that was processed immediately after exposure to EMF (87 kHz, 1.05 kW, 1.4–1.7 mT), we did not notice any negative, cytotoxic effect on cell viability (Fig. 14). Although there was a slight decrease in viability by 4% in the case of NHA cells or an increase of 7–8% for HDFa and SH-SY5Y, it was not a significant decrease. The set parameters of the WPT system did not have a negative effect on the analyzed cells.

In the second part of the experiment, we let the cells regenerate for 30 h. During this period, there may have been a negative, cumulative effect of EMF exposure on cell viability compared to the control group. Analysis of the effect of radiation produced at a frequency of 87 kHz on cell viability 30 h after regeneration (Fig. 15A) did not show any significant negative effect. Only slight variations were noted within the analysis of the individual tested groups (pulsed EMF 6 × 10 min, continuous EMF 1 × 60 min). All cells exposed to pulsed radiation (6 × 10 min) showed a slight, statistically insignificant increase in measured values by an average of 4%. No analyzed change in cell viability was significant, either for healthy or tumor cells or for continuous exposure (1 × 60 min).

The viability analyses of tumor cells exposed to continuous EMF (1 × 60 min) were interesting. Radiation had no effect on healthy HDFa and NHA cells (viability was 103% for HDFa and 100% for NHA). Viability was affected in SH-SY5Y and T98G tumor cells. For neuroblastoma, SH-SY5Y there was a 10% decrease in cell viability, on the contrary for glioblastoma, there was a 10% increase in viability. For now, we can only hypothesize that neuroblastomas are more sensitive to the given radiation compared to aggressive forms of glioblastoma, in which, on the contrary, this radiation promoted expansion.

In the third part of the experiment, we analyzed the effect of continuous exposure 1 × 60 min generated at an increased frequency of 207 kHz (Fig. 15B). In healthy cells (HDFa and NHA), we observed a drop in viability (to 96% and 92%, respectively) with continuous radiation 1 × 60 min. We recorded a drop in viability to 92% in NHA even with continuous exposure to EMF for 60 min. All these decreases were not significant and were within the calculated deviations.

In the case of tumor cell lines (SH-SY5Y and T98G), the observations were already more interesting. The SH-SY5Y neuroblastoma line showed a significant decrease in cell viability, especially with continuous 60 min irradiation (to 66%). A decrease was also recorded after 30 min of irradiation (to 85%). This drop is within the deviation.

The results of measurements of T98G glioblastoma cells showed the opposite effect on cell viability. With 30 min of continuous irradiation, there was an increase in viability to 113%, and with 60 min of radiation up to 117%. Similarly, as was the case with 60 min of continuous radiation at a frequency of 87 kHz, neuroblastoma cells are significantly more sensitive, and their cell viability is negatively affected during prolonged exposure. We can assume that if the patient has a glioblastoma, then most likely a prolonged stay in an environment with increased exposure will accelerate the development of this cancer. On the other hand, it is necessary to consider the fact that these are simulation conditions that far exceed the range of standards given by ICNIRP 2010 and 2020.

### Effect of EMF on cellular cytotoxicity

**Figure 16:**
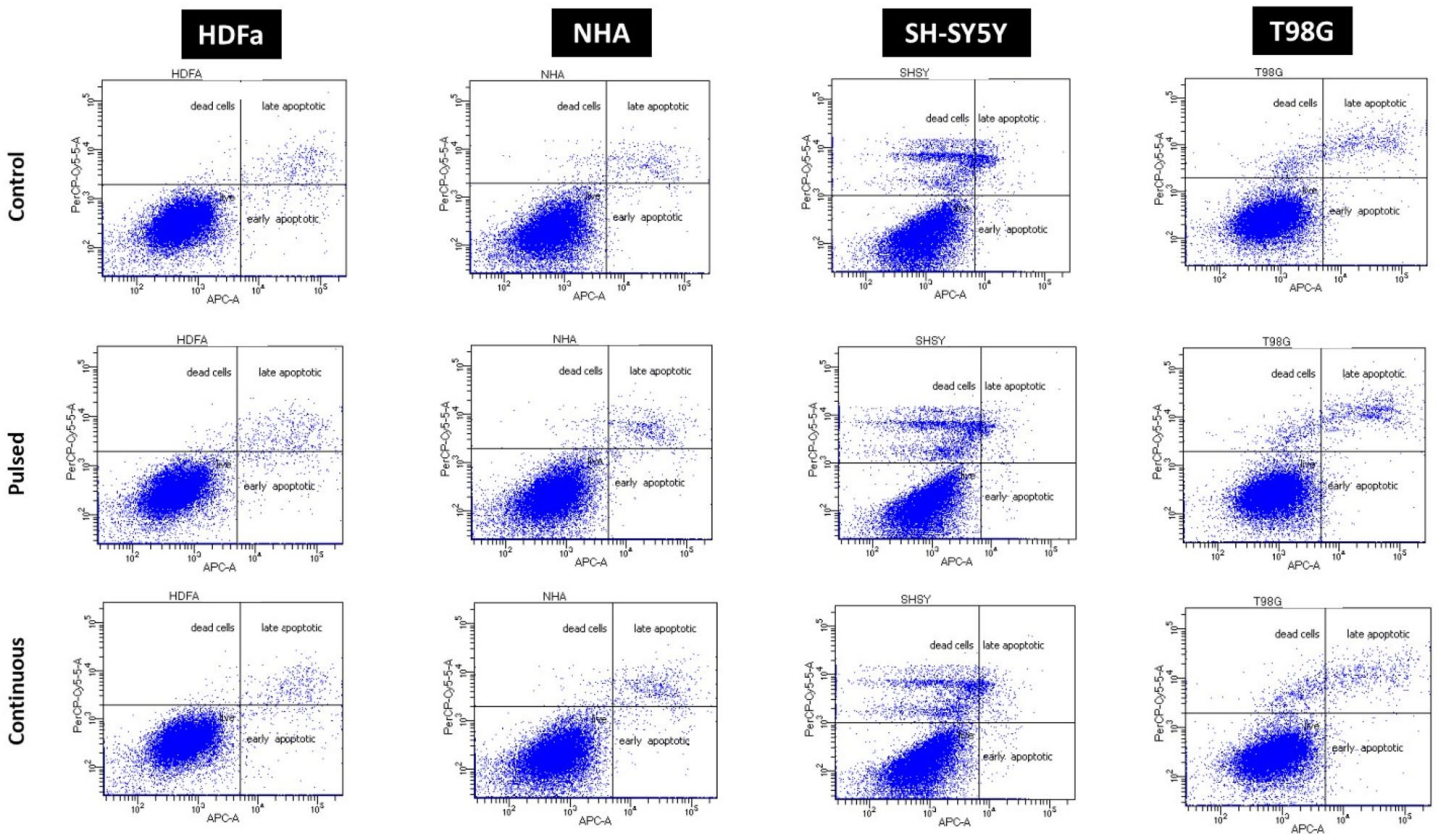
The ratio of live, apoptotic, and necrotic HDFa, NHA, SH-SY5Y, and T98G cells after regeneration 30 h after EMF exposure. The ratio was determined based on the results of flow cytometry and Annexin V assay. Cells were exposed to EMF parameters of 87 kHz, 1.4–1.7 mT, and 1.05 kW during pulsed exposure (6 × 10 min) and continuous EMF exposure (1 × 60 min). The control group was not exposed to EMF.

We determined the ratio of live, apoptotic, and necrotic cells by flow cytometry and Annexin V analysis. We evaluated all four cell types. We compared exposed cells exposed to pulsed EMF (6 × 10 min) and continuous EMF (1 × 60 min) with a control group that was not exposed to EMF. The observed results of the analysis are summarized in Fig. 16 and Table 2.

**Table 2:** The effect of EMF on the death of cells exposed to different irradiation conditions (pulsed EMF 6 × 10 min, continuous EMF 1 × 60 min).

		Living cells	Early apoptic	Late apoptic	Dead cells
HDFa	Control	96.20	1.70	1.80	0.30
	Pulsed	94.90	2.50	2.20	0.40
	Continuous	95.30	2.60	1.90	0.20
NHA	Control	95.20	2.30	1.90	0.60
	Pulsed	94.30	2.50	2.60	0.60
	Continuous	94.00	2.80	2.70	0.50
SH-SY5Y	Control	84.20	1.80	3.40	10.60
	Pulsed	82.00	1.00	2.10	14.90
	Continuous	83.90	1.20	2.30	12.60
T98G	Control	92.10	2.60	3.10	2.20
	Pulsed	90.50	3.20	4.10	2.20
	Continuous	91.60	2.90	2.80	2.70

The cells were regenerated for the following 30 h. Samples were analyzed by flow cytometry based on the Annexin V assay.

Under the analyzed conditions, we did not observe increased cell death in any cell type. The “live cells” group represents more than 90% of the total number of cells analyzed (for HDFa it is more than 95%, for NHA it is more than 94% and for T98G it is a population of more than 90% cells). SH-SY5Y have the lowest % number of “live cells” at an average of 83%. This value is due to the slightly increased confluence of the cell culture due to the favorable cultivation conditions. SH-SY5Y, after reaching high confluence (100%; see the image of cell morphology), does not slow down the metabolism or the division process, because it is a tumor cell line. Cells lose their ability to adhere to the culture flask and this also leads to an increased number of cells in the process of cell death (the other three analyzed phases).

The “dead cells” group represents the lowest percentage representation (less than 0.6%) among the four analyzed cellular processes in healthy cells (HDFa and NHA). This indicates that the cells had suitable conditions for growth, and the generated EMF did not negatively affect them.

The percentage of cells in the phase of regulated cell death—apoptosis (“early” and “late” phase) is lower than 2.8% of the total number in both types of healthy cells.

For the tumor line, T98G, the percentage of cells in the “dead cell” phase is less than 2.7%. The other two processes analyzed in T98G (“early apoptosis” and “late apoptosis”) are only slightly increased compared to the “dead cells” group.

The tumor cell line, SH-SY5Y, is noteworthy because in the “dead cells” phase it is the second group with the highest percentage representation within the four cellular processes analyzed (“live cells”, “early apoptosis”, “late apoptosis” and “dead cells”), from 11 to 15%. While pulse irradiation achieved the highest percentage representation (almost 15%) compared to the control group (almost 11%). We attribute this increase to the tumor phenotype of the cells and not to the negative influence of the EMF generated by our WPT system, as the SH-SY5Y control group showed a very similar trend to the irradiated cells. The percentage of cells in the process of apoptosis (“early” and “late” phase) is significantly lower than the “dead cell” phase, at less than 3.4%. This observation only confirms the fact that SH-SY5Y cells lose adherence and die after reaching high confluence. These findings are also confirmed by the analysis of morphology through light microscopy (Fig. 20), where the confluence of SH-SY5Y is the highest compared to the other cell lines.

### Effect of EMF on cellular genotoxicity

**Figure 17:**
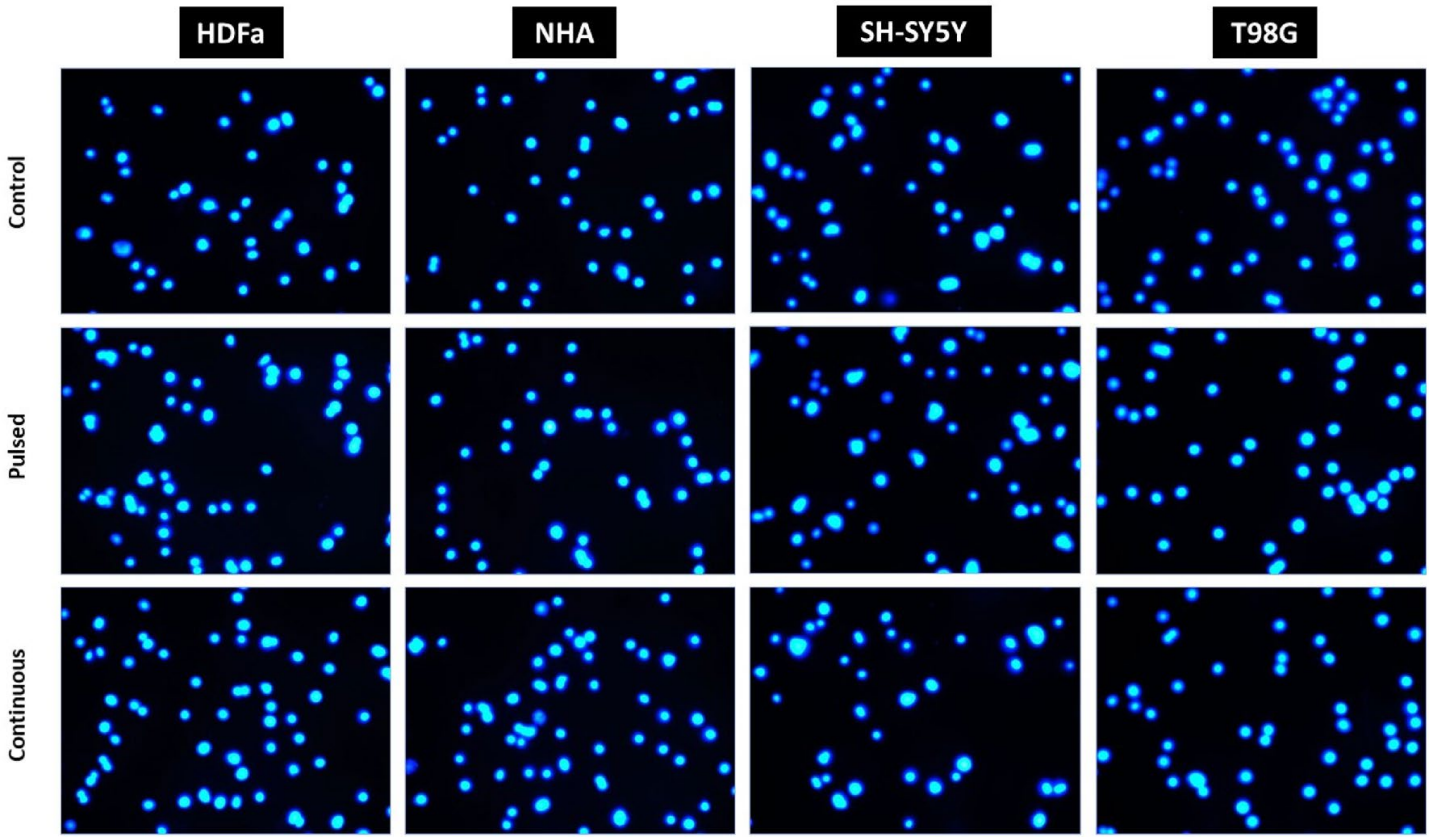
Effect of EMF on genotoxic changes and DNA damage in cells exposed to different irradiation conditions (pulsed EMF 6 × 10 min, continuous EMF 1 × 60 min). The cells were regenerated for the following 30 h. The samples were analyzed by fluorescence microscopy at magnification 200×.

A Comet assay was performed to demonstrate the genotoxic effect of EMF generated by the WPT system. Under the analyzed conditions, we did not observe any DNA breaking points (comets) in any cell type (Fig. 17).

The EMF generated at our parameters (87 kHz, 1.4–1.7 mT, 1.05 kW) does not cause detectable DNA damage identified by the comet tail in either test group (pulsed EMF 6 × 10 min, continuous EMF 1 × 60 min) compared to the control which has not been exposed. Since no genotoxic changes were analyzed, the DNA of the cells remained intact, and the effect was not manifested even in the mitotic activity of the cells. Direct evidence of this is the increased cell confluence.

### Effect of EMF on cellular oxidative stress

The assay was performed simultaneously with the measurement of cell viability. In one well, we first determined the cell viability by fluorescence measurement, and then after removing the culture medium, lysing the cells, and adding the substrates, we measured the luminescence signal in individual samples. Based on the calibration curve, we determined the amount of GSH in the control and irradiated samples. Results are given in units of µmol/L.

**Figure 18:**
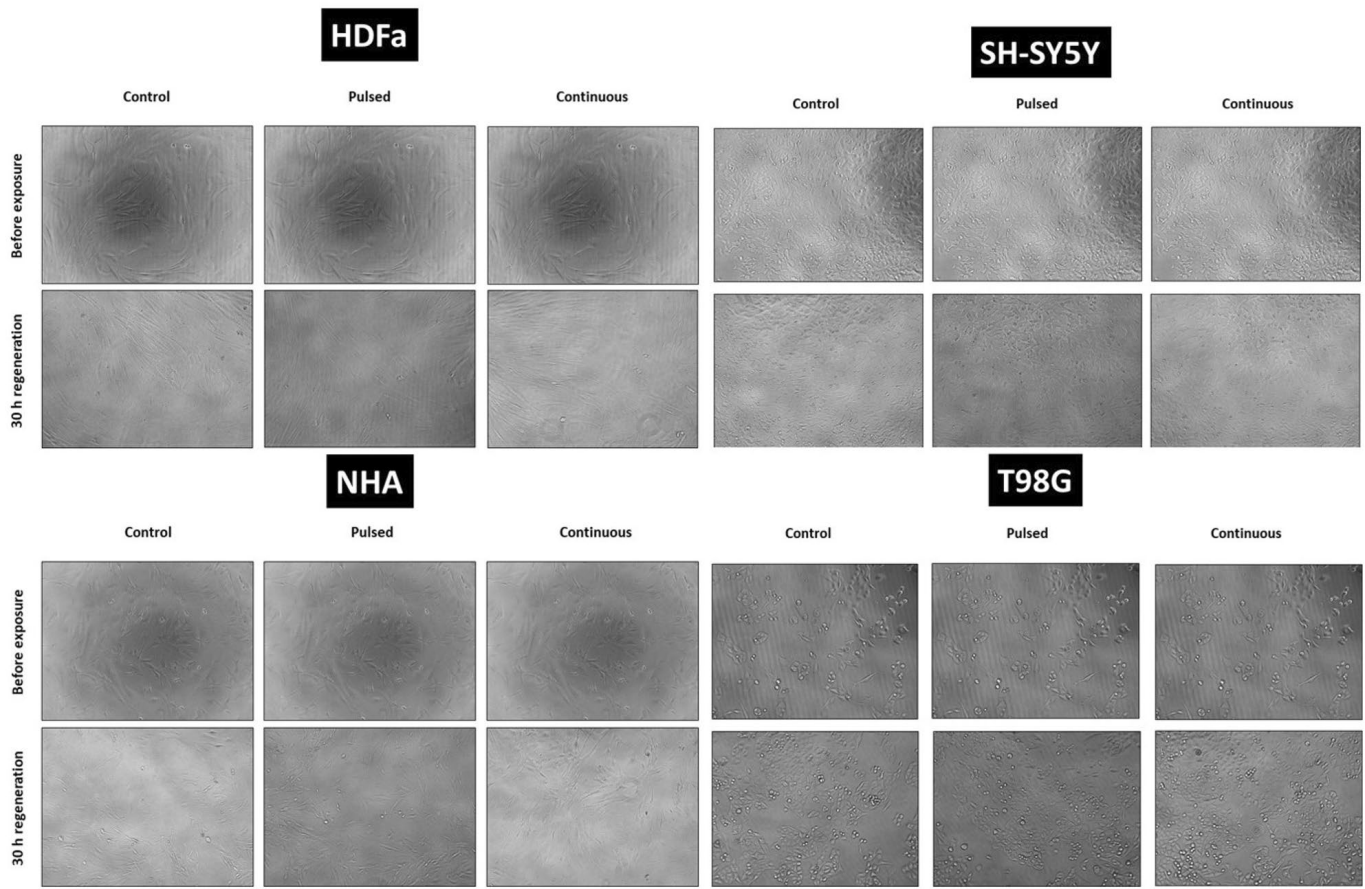
Cell morphology of human dermal fibroblasts (HDFa) and human neural cells (NHA, T98G, SH-SY5Y) before EMF exposure and after EMF exposure (pulsed/continuous EMF) and subsequent regeneration for 30 h. Control cells were not exposed to EMF (first column), cells exposed to pulsed radiation (87 kHz, 6 × 10 min, second column), and cells exposed to continuous radiation (87 kHz, 1 × 60 min, 3rd column). Representative images were taken by phase contrast at 100× magnification.

In the first part of the experiment, we observed cell viability using light microscopy (Fig. 18). We did not observe any visible changes in the morphology or adherence ability of cells exposed to EMF generated by the WPT system at operating parameters (87 kHz, 1.4–1.7 mT, 1.05 kW) compared to the control.

The cells were not affected, or inhibited mitotic activity, because, after 30 h of regeneration under standard conditions, we observed an increased confluence of cells from 20 to 30% (after exposure) to 40–50% (after regeneration). The exception is SH-SY5Y cells, where cell confluence increased from 55% (after exposure) to almost 100% (after regeneration). Cell confluence as well as preserved morphology was comparable to the control group in the exposed cells. We did not observe an increased number of dead cells in either cell type.

**Figure 19:**
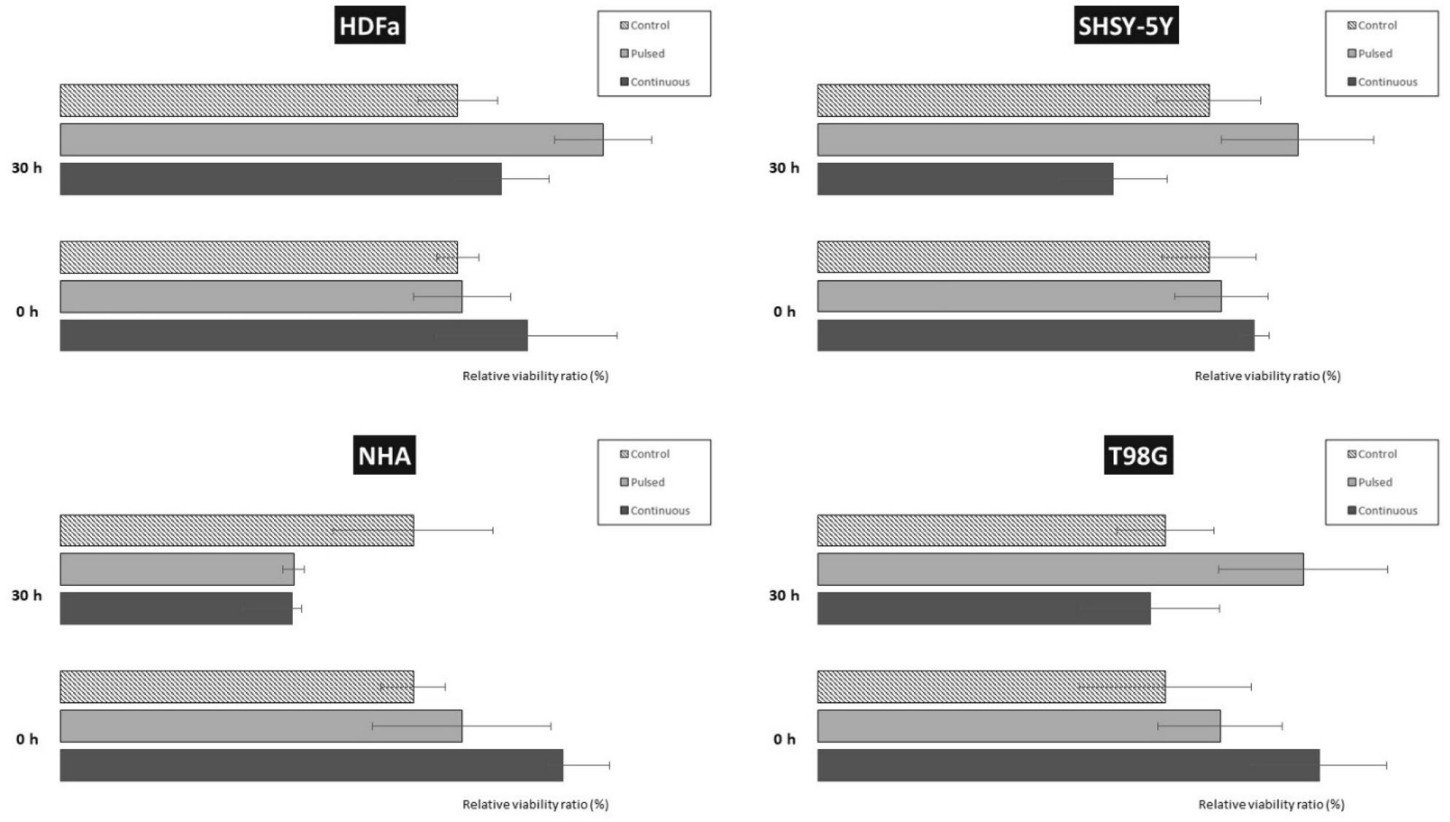
Effect of EMF exposure on the viability of HDFa, NHA, SH-SY5Y, and T98G cells. Cell viability was analyzed by fluorescence measurement. Cells were exposed to EMF (87 kHz, 1.4–1.7 mT, 1.05 kW) for pulsed exposure (6 × 10 min) and continuous exposure (1 × 60 min). The control group represents cell viability of 100%.

In the second part, we analyzed cell viability by fluorescence measurement. The results showed no significant changes in the viability of cells analyzed immediately after irradiation compared to cells after regeneration for 30 h. Although the variability within the individual groups (time-lapse, cell type) is significant, it is caused by the measurement (Fig. 19).

The results indicate a slight increase in cell viability in cells exposed to pulsed radiation both at time 0 and at 30 h after regeneration. This increase ranges from 102 to 138% in both healthy cells and tumor cells.

The upward trend was maintained in cells exposed to continuous EMF exposure. The results show a slight increase in cell viability (110–130%) both at time 0 and after 30 h of regeneration (an increase in the range of 105–110% for healthy cells). A slight decrease in viability was observed in SH-SY5Y and T98G tumor cell lines exposed to continuous EMF exposure. The decrease in relative viability compared to the control was 5–10%, however, it is negligible.

**Figure 20:**
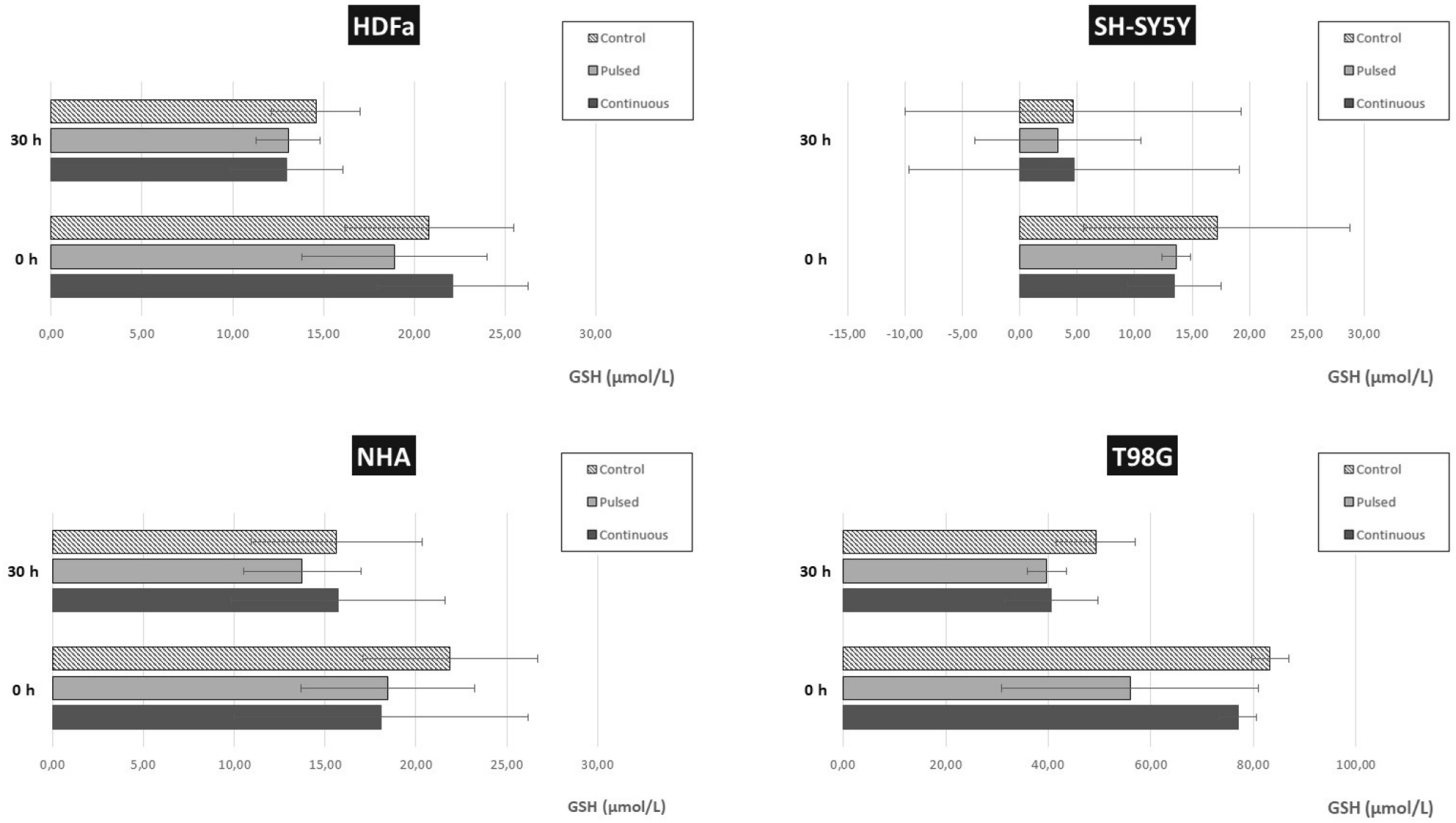
Determination of the level of oxidative stress and the total amount of reduced glutathione (GSH) in HDFa, NHA, SH-SY5Y, and T98G cells. The level of GSH was determined by luminescence measurement followed by extrapolation to the calibration curve. Cells were exposed to EMF (87 kHz, 1.4–1.7 mT, 1.05 kW) for pulsed exposure (6 × 10 min) and continuous exposure (1 × 60 min). The control group was not exposed to EMF and represents 100%.

In the third part, we determined the level of oxidative stress of cells through luminescence measurement. We determined the total amount of reduced glutathione (GSH) by measuring the luminescence in the control and exposed samples and subsequent extrapolation to the calibration curve. There was no significant change (either increase or decrease) compared to the control group even with either type of irradiation (pulsed or continuous). Although the variability of the determined total GSH is significant, this change is not significant (Fig. 20).

In healthy cells (HDFa and NHA), the value of total GSH in the control group (22 µmol/L and 16 µmol/L), as well as the group exposed to pulsed radiation (19 µmol/L and 13 µmol/L), is very comparable to both in the samples analyzed immediately after irradiation or after regeneration for 30 h. In the case of continuous radiation, these values vary between 13 and 22 µmol/L for HDFa and 16–18 µmol/L for NHA cells.

In the case of SH-SY5Y tumor cells, we observe a trend similar to NHA cells. In the group in which total GSH was determined immediately after irradiation, we measured a value of total GSH of 17 µmol/L compared to both irradiated groups, which showed a value of total GSH of 13 µmol/L. An interesting observation is in the group of SH-SY5Y cells after regeneration for 30 h. The standard deviation is significantly higher than the measured value of total GSH itself. These cells had the highest confluence compared to the other three cell types, but the measured level of total GSH was the lowest.

T98G tumor cells show significantly higher total GSH concentrations compared to other cell types. In the control group itself, we measured the amount of total GSH up to 83 µmol/L in samples analyzed immediately after irradiation and 49 µmol/L after 30 h regeneration. In the irradiated groups, the total amount of GSH varied from 95 to 131 µmol/L. Why these cells have such a high level of GSH is worth further analysis.

## Discussion

People are exposed to several natural sources of electromagnetic radiation (EMR) and EMF, such as the sun, cosmic radiation, storms, and atmospheric discharges. However, the number of artificial EMR and EMF sources has increased enormously in recent decades. Mobile phones, tower antennas, power stations, transmission lines, radars, televisions, and other electronic devices have become part of the daily life of a large part of the population^24^. Therefore, the interest in monitoring either short-term or long-term effects of EMF on human health, especially when it comes to high exposure, is justified and desirable^25^. Current opinions on the impact of EMR and EMF are debatable.

Although there are several beneficial, therapeutic applications of EMF e.g. in tissue engineering or in the field of wound healing^26,27^, several publications point to a negative impact^28,29^. Headaches, fatigue, concentration problems, depression, sleep disorders, and immune and hormonal disorders are just a short list of the negative impact of short-term exposure to EMF (whether radio frequency or low frequency). The effect of long-term EMF exposure is primarily linked to the development of oncological diseases^10,30,31^.

EMF and the generated electromagnetic waves affect many potential targets in the living organism. The human organism and its biological functions are controlled by electrical potentials and currents– transmissions of excitement in the nervous and neuromuscular systems, blood flow, or membrane transport at the cellular level^32,33^. Artificially created EMF affects the electrochemical homeostasis in cells, thus posing a risk^34^. Therefore, to fully understand the effect of artificial EMF on the organism, it is necessary to know the mechanisms of action. Therefore, many experimental and comparative studies were carried out on various models, either animal or cell lines, but also population studies.

The aim of this study was to test a newly built device prototype for a wireless charging system and investigate the effect of generated EMF and electromagnetic waves on changes in cell biology in vitro. We tested four cell lines simultaneously. Two cell lines represented normal cells (HDFa and NHA) and two represented tumor cell lines (SH-SY5Y and T98G). The choice of cell lines offered biological variability, thanks to which we obtained more comprehensive results about the effect of EMF exposure on a living system. Dermal fibroblasts were chosen primarily because they are the first to be exposed to EMF. HDFa cells create and maintain multiple, anatomically diverse, connective tissues. They have a wide range of biological properties such as mechanical resistance, elasticity, excellent regeneration ability, and production of extracellular matrix. Fibroblasts can form a transient and contractile myofibroblast phenotype in response to tissue damage and respond by secreting soluble mediators such as cytokines, growth factors, and metabolites^34^.

EMF has been shown to have significant effects on basic neural functions and synaptic plasticity^35^. Nerve cell lines were chosen because they are relatively sensitive, conduct electrical impulses in the nervous system, and their regenerative capacity is limited.

Astrocytes play an irreplaceable role in the CNS. The number of NHAs significantly exceeds the number of neurons in the human brain. Astrocytes are involved in maintaining the homeostasis of ions, neurotransmitters, and other molecules. At the same time, they are involved in the formation and elimination of synapses and are associated with antioxidant defense, thereby protecting neurons sensitive to an excess of reactive oxygen/nitrogen forms and oxidative stress^36^.

The neuroblastoma line SH-SY5Y is among the most used cell lines in neuroscience. SH-SY5Y represent an excellent model for testing neurotoxicity^37,38^ similar to the TG9G cell line. These are cells of human glioblastoma multiforme, the most widespread and malignant form. T98G have a spherical, polygonal, and fibroblastic shape^39^.

Akbarnejad et al. confirmed that exposure of T89G cells to EMF exposure (100 Hz, 100 G) combined with treatment of cells with different molar concentrations of temozolomide increases its toxicity on glioblastoma cells^40^. The group von Niederhäusern et al. investigated the effect of exposure of SH-SY5Y cells to RF-EMF exposure (935 MHz, 4 W/kg, 24 h) on neural differentiation and mitochondrial respiration. And although the exposure did not show a significant effect on the differentiation of SH-SY5Y, they noted impairment of mitochondrial respiration in glucose-deprived cells^41^.

The expansion of the use of wireless devices and communications has brought with it exposure to EMF. Several studies have provided valuable information on the effect of EMF on living organisms, animals, or cell models at different EMR and EMF parameters. The group of Kim et al. published several findings on the negative impact of EMF exposure on the CNS, on neuronal damage, demyelination, calcium homeostasis, disorders of neurotransmitter release, changes in postsynaptic structure, and limited neurite growth^42–45^. Masoudi-Khoram and Abdolmaleki's group revealed that exposure to EMF (50 Hz, 20 mT) remarkably decreased the viability of breast cancer cells compared to a control group not exposed to EMF^46^. It has also been suggested that, when used in low doses, EMF may have therapeutic properties. Gao et al.^47^ found that extremely low-frequency EMF (50 Hz, 1 mT) promoted cognitive function and hippocampal neurogenesis in rat models of cerebral ischemia.

In this study, we observed the effect of short-term EMF exposure on the complex cell biology of four exposed cell lines and compared them with control cells not exposed to EMF. We have provided background information on the possible biological effects of acute EMR and EMF exposure in the RF range and the implications for living systems in vitro. We analyzed cell morphology, cytoskeletal integrity, cell viability, the effect on cell cytotoxicity, genotoxicity, and DNA integrity, as well as the response to cellular oxidative stress. Furthermore, we observed both the immediate effect and the cumulative effect of EMF irradiation (30 h regeneration).

Based on the set parameters (87 kHz, 1.4–1.7 mT, 1.05 kW), we did not notice any changes in morphology, phenotype, and adherence in any cell type (Fig. 12). Exposure to EMF (pulsed or continuous) did not play a role in this case. In our case, exposure to EMF did not even disrupt mitotic activity, cell confluence increased throughout the regeneration period (30 h).

The immunocytochemical analysis did not confirm intracellular reorganization of the cytoskeleton in any cell type after regeneration (30 h) under standard culture conditions (Fig. 13). The results of the analysis were comparable with the control groups (whether it was pulsed or continuous exposure). We did not observe a significantly negative, cytotoxic effect on cell viability and energy metabolism of cells. MTT analysis did not show statistically significant changes in cell viability either in cases immediately after irradiation (continuous EMF) or after regeneration for 30 h (pulsed and continuous EMF). A significant decrease in viability was not observed even after 30 h of regeneration, in the case of continuous EMF exposure with a 2.5× increased frequency (207 kHz, 1.3–1.5 mT, 1.01 kW) of the WPT system. A negative effect of EMF (1.762 GHz, 8 W/kg) was also not observed by the group of Jin et al., either on viability or on DNA double-strand breaks (induced by physical and chemical agents). On the contrary, such EMF promoted its repair, did not induce apoptosis or necrosis^48^.

We did not observe any increase in cell death under our test conditions. The determined ratio of live, apoptotic, necrotic, and dead cells, evaluated using flow cytometry and Annexin V assay, was comparable in both the group exposed to pulsed EMF, and the group exposed to continuous EMF after the 30 h regeneration period. The group of live cells made up on average more than 90% of the total number of analyzed cells (Fig. 16). There is some controversy here. Many studies report that non-ionizing, non-thermal, extremely low-frequency EMF can modulate apoptosis. However, the results of other studies differ and there is an opinion that statistically significant changes in apoptosis are not observed^49^. However, they did not observe a statistically significant difference in the study by Martinelli et al., when exposed to EMF (915 MHz) on cardiac cells, their structural tissue integrity, and the expression of apoptotic genes^50^.

No damage or breaks in DNA were observed. The cumulative, genotoxic effect of EMF (after 30 h regeneration) was not manifested in any cell type when exposed to pulsed or continuous EMF. We determined the effect of EMF on cellular oxidative stress and cellular viability simultaneously after regeneration for 30 h. Fluorescence analysis of cell viability of selected cell lines did not show any significant negative changes. The results showed a slight increase in viability in cells exposed to pulsed radiation (both 0 and 30 h after regeneration). This increase ranges from 102 to 138% in both normal cell lines and tumor cells.

We further observed the cellular stress response in response to EMF exposure. Some studies suggest that oxygen radical levels increase more significantly after short-term EMF exposure than after long-term EMF exposure (≥ 12 h)^51,52^. The level of oxidative stress of the cells was determined in this case by luminescence measurement. We determined the total amount of reduced glutathione (GSH) by measuring the luminescence in the control and exposed samples. The results were then extrapolated to the calibration curve. The values of total measured GSH varied depending on the cell type. T98G tumor cells show significantly higher total GSH concentration values compared to other cell types. However, we did not find a significant difference. Although we determined the total concentration of GSH (µmol/L) in individual cells, it would be good to normalize the measurements either to the number of cells or to the total amount of protein. However, the kit manufacturer does not offer this option. The comparison of measured GSH values between individual cell lines (with different achieved confluence) is therefore complicated. We are also inclined to say that short-term exposure to EMF triggers antioxidant processes. Because the antioxidant system is not yet exhausted, levels of oxidative stress do not match observations with long-term EMF exposure^53^.

Our results demonstrated that the tested parameters (which were deliberately higher than the recommended value) of our WPT device generating EMR and EMF are relatively safe and harmless to normal cell lines. The use of the new WPT device should not pose any risk as it did not cause significant cytotoxic and genotoxic changes in vitro. However, this study was only performed on cell lines, we cannot report clinical implications.

## Conclusion

In summary, exposure to EMF generated by the WPT device at operating parameters of 87 kHz, 1.05 kW and 1.4–1.7 mT is safe for HDFa, NHA, SH-SY5Y, and T98G cell lines. Changes were not observed even with EMF exposure with device operating parameters of 207 kHz, 1.01 kW and 1.3–1.5 mT. Whether the cells were exposed to pulsed or continuous EMF did not play a significant role either.

## Data Availability

All data generated or analysed during this study are included in this published article.
